# A Nucleic Acid‐Based LYTAC Plus Platform to Simultaneously Mediate Disease‐Driven Protein Downregulation

**DOI:** 10.1002/advs.202306248

**Published:** 2024-01-22

**Authors:** Yangyang Huang, Xujiao Zhou, Yirou Zhang, Miao Xie, Fujun Wang, Jingcan Qin, Han Ye, Hong Zhang, Chuan Zhang, Jiaxu Hong

**Affiliations:** ^1^ School of Chemistry and Chemical Engineering Frontiers Science Center for Transformative Molecules Shanghai Key Laboratory for Molecular Engineering of Chiral Drugs Shanghai Jiao Tong University Shanghai 200240 P. R. China; ^2^ Department of Ophthalmology and Vision Science Shanghai Eye, Ear, Nose and Throat Hospital Fudan University Shanghai 200030 P. R. China; ^3^ Department of Radiology Changhai Hospital Naval Medical University Shanghai 200433 P. R. China; ^4^ Department of Ophthalmology the Affiliated Hospital of Guizhou Medical University Guiyang 550025 P. R. China; ^5^ Shanghai Engineering Research Center of Synthetic Immunology Shanghai 200032 China

**Keywords:** LYTACs, n‐AMD, nuclear acid hydrogel, protein degradation, siRNA

## Abstract

Protein degradation techniques, such as proteolysis‐targeting chimeras (PROTACs) and lysosome‐targeting chimeras (LYTACs), have emerged as promising therapeutic strategies for the treatment of diseases. However, the efficacy of current protein degradation methods still needs to be improved to address the complex mechanisms underlying diseases. Herein, a LYTAC Plus hydrogel engineered is proposed by nucleic acid self‐assembly, which integrates a gene silencing motif into a LYTAC construct to enhance its therapeutic potential. As a proof‐of‐concept study, vascular endothelial growth factor receptor (VEGFR)‐binding peptides and mannose‐6 phosphate (M6P) moieties into a self‐assembled nucleic acid hydrogel are introduced, enabling its LYTAC capability. Small interference RNAs (siRNAs) is then employed that target the angiopoietin‐2 (ANG‐2) gene as cross‐linkers for hydrogel formation, giving the final LYTAC Plus hydrogel gene silencing ability. With dual functionalities, the LYTAC Plus hydrogel demonstrated effectiveness in simultaneously reducing the levels of VEGFR‐2 and ANG‐2 both in vitro and in vivo, as well as in improving therapeutic outcomes in treating neovascular age‐related macular degeneration in a mouse model. As a general material platform, the LYTAC Plus hydrogel may possess great potential for the treatment of various diseases and warrant further investigation.

## Graphical abstract

A LYTAC Plus hydrogel that combines gene silencing and protein degradation capabilities is engineered through nucleic acid self‐assembly. By simultaneously downregulating the VEGFR‐2 and ANG‐2 levels, LYTAC Plus hydrogel designed for alleviating neovascular age‐related macular degeneration (n‐AMD) shows a better therapeutic efficacy compared to the conventional approach in the mouse model, indicating its potential as a versatile platform for disease treatments.

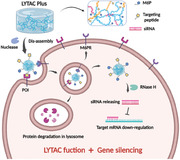

## 1 Introduction

A large variety of diseases have been identified as being induced by the mutation or accumulation of specific proteins, which has led to the development of various protein degradation tools for disease treatments.^[^ ^1^ ^]^ The most famous one among them, proteolysis‐targeting chimeras (PROTACs), relies on recruiting ubiquitin ligases to the intracellular target proteins to promote the degradation of disease‐driven proteins by proteasome machinery, enabling many “undruggable” proteins to become therapeutic targets.^[^ ^2^ ^]^ To date, tens and hundreds of PROTAC drugs have been developed for clinical purposes, and some have advanced to phase II.^[^ ^3^ ^]^ Similar to PROTAC technology, other innate cellular degradation machineries have been employed to degrade the protein of interest (POI), leading to the emergence of autophagosome‐targeting chimeras (AUTACs), lysosome‐targeting chimeras (LYTACs), and ribosome‐targeting chimeras (RIBOTACs).^[^ ^4^ ^]^ Unlike PROTACs and AUTACs, which mainly target intracellular proteins, LYTACs generally focus on capturing secretory and membrane‐associated proteins for degradation, such as growth factors, disease‐associated receptors, and cytokines,^[^ ^5^ ^]^ which make up approximately 40% of the human proteome and are important therapeutic targets for many diseases.^[^ ^6^ ^]^ Unlike PROTACs, which utilize the proteasomal pathway, LYTACs rely on the recognition of lysosome‐targeting receptors (LTR), such as cation‐independent mannose‐6 phosphate receptor (CI‐M6PR, also known as IGF2R), to actively transport the POIs into the lysosomes for degradation.^[^ ^5^, ^7^ ^]^

A typical LYTAC design contains one or multiple M6PR binders and the targeting motifs of POI.^[^ ^5^, ^8^ ^]^ Similarly, other surface transporting receptors, such as the GalNAc‐N‐acetylgalactosamine receptor, have also been explored in LYTAC design, showing a more tissue‐specific feature than the conventional M6P‐M6PR system.^[^ ^9^ ^]^ Despite these advances, challenges remain in all protein degradation techniques. In general, the therapeutic benefits are highly related to the effectiveness of the target protein degradation. Compared to PROTAC molecules, the overall efficiency of LYTAC‐based POI elimination is typically lower, thus casting a shadow on its future clinical use. It is noteworthy that many diseases are related to multiple factors, which means that a single protein degradation may not be sufficient to meet clinical demands. Therefore, there is an urgent need to design new and powerful LYTAC materials that can improve their effectiveness and functionality for disease treatment.

**Figure 1:**
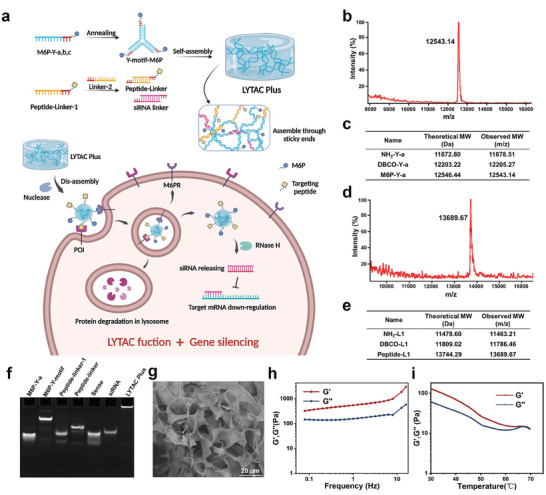
Characterizations of the LYTAC Plus hydrogel. a) The mechanism of LYTAC Plus for simultaneous membrane protein degradation and gene silencing. b,c) Representative MALDI‐TOF spectrum and analysis of DNA after M6P modification. d,e) MALDI‐TOF spectrum and analysis of DNA after peptide modification. f) Self‐assembly of M6P‐Y‐motif, peptide‐linker, siRNA, and LYTAC Plus gel characterized by 10% native PAGE. g) SEM image of freeze‐dried LYTAC Plus gel. Scale bar: 20 µm h) Modulus (*G′* and *G″*) of LYTAC Plus gel from frequency sweep rheological tests. i) Modulus (*G′* and *G″*) from temperature‐ramp rheological tests of the LYTAC Plus gel.

To combat multi‐factor driven diseases, the most commonly used strategy is combinatory treatment, which is supported by thousands of studies on the synergistic use of different therapeutic modules,^[^ ^10^ ^]^ as well as hundreds of clinical investigations employing two different therapeutics.^[^ ^11^ ^]^ To create a more powerful LYTAC candidate, we envision that incorporating other protein silencing strategies into the LYTAC construct may allow us to enhance its therapeutic efficacy and achieve synergistic effects in disease treatments. Thus far, various target protein silencing approaches,^[^ ^12^ ^]^ such as antisense technology,^[^ ^13^ ^]^ RNA interference,^[^ ^14^ ^]^ target gene editing and disruption,^[^ ^15^ ^]^ have been widely explored for medical purposes, some of which have been successful in clinical use. For example, self‐assembled nucleic acid hydrogels have been developed as new vehicles for functional RNA therapeutic delivery owing to their easy synthesis, excellent biocompatibility, and programmable self‐assembly.^[^ ^16^ ^]^ With the maturity of DNA/RNA modification, lysosome‐targeting and POI‐binding motifs can now be readily equipped with nucleic acid hydrogels, enabling the engineering of next‐generation LYTAC materials, which we term LYTAC Plus (**Figure** **1**a).

As a proof‐of‐concept study, we specifically designed a LYTAC Plus hydrogel for treating neovascular age‐related macular degeneration (n‐AMD), one of the most common causes of irreversible visual loss worldwide.^[^ ^17^ ^]^ Although anti‐VEGF agents have been widely used as the first‐line drug for n‐AMD treatment and show outstanding clinical benefits,^[^ ^18^ ^]^ a certain patients do not respond well to those anti‐VEGF therapeutics, and more than 50% of n‐AMD patients build up resistance to the drug after 1 year of sustained anti‐VEGF therapy.^[^ ^19^ ^]^ As a multi‐factor driven disease,^[^ ^19b^ ^]^ overexpression of angiopoietin 2 (ANG‐2) has been verified as an important driving force in the progression of n‐AMD^[^ ^20^ ^]^ by destabilizing blood vessels.^[^ ^21^ ^]^ Therefore, simultaneously reducing the levels of both VEGF/VEGFR and ANG‐2 may significantly promote the therapeutic outcome of n‐AMD treatment.^[^ ^22^ ^]^

Therefore, we modified one of the component DNA strands that assemble into Y‐shape and double‐stranded DNA cross‐linker building blocks with M6P moiety and VEGFR binding peptide (VEGF_125‐136_) respectively, enabling the hydrogel with LYTAC capability. Furthermore, anti‐ANG‐2 siRNAs were also designed as cross‐linkers with overhangs that could hybridize with M6P‐equipped Y‐motifs by Watson‐Crick base pairings, resulting in the formation of LYTAC Plus hydrogel. Upon vitreous injection into the eye, the LYTAC Plus hydrogel is gradually degraded into smaller particles by DNase I, allowing its efficient uptake by surrounding cells. With both LYTAC and gene silencing capabilities, LYTAC Plus hydrogel can rapidly transport surface VEGFR into lysosomes for degradation. The siRNA released from LYTAC Plus further downregulates ANG‐2 expression through the RNAi mechanism. In a mouse model of n‐AMD, we demonstrated that simultaneously reducing the levels of VEGFR‐2 and ANG‐2 by LYTAC Plus hydrogel indeed improved the therapeutic effect compared to the control groups, as evidenced by small leakage fluorescence, reduced choroidal neovascularization (CNV) areas and width, as well as enhanced electrophysiological function of the inner retina.

## 2 Results and Discussion

### 2.1 Preparation and Characterization of LYTAC Plus

Nucleic acid hydrogels have shown excellent performance in both drug and siRNA deliveries due to their characteristics of easy assembly, strong modifiability, and high biocompatibility.^[^ ^23^ ^]^ Therefore, we chose to use nucleic acid hydrogels as an integrated system for both the LYTAC function and the Plus function, which is gene silencing. To endow the nucleic acid hydrogel LYTAC capability, we modified the component DNA strands of Y‐motif with the M6P molecule and one DNA linker strand with targeting peptide by click reactions. To achieve this goal, we synthesized the azide‐modified M6P (M6P‐N_3_) following the previous literature, with some modifications.^[^ ^24^ ^]^ The synthesis of M6P‐N_3_ involved a 10‐step reaction process, as shown in Scheme S1 (Supporting Information). Intermediate products and the final compound were confirmed using hydrogen and carbon nuclear magnetic resonance spectra (^1^H‐NMR and ^13^C‐NMR, respectively) as shown in Figure S1–S16 (Supporting Information). The mass spectrometry analysis revealed a mass/charge ratio (m/z) value of 342.07 for [M‐H]^−^ ions, indicating the successful synthesis of M6P‐N_3_ (Figure S16, Supporting Information). To conjugate with either M6P‐N_3_ or peptide‐N_3_, DBCO moieties were modified on amine‐terminated DNA (NH_2_‐DNA) by a well‐established NHS ester reaction (Scheme S2, Supporting Information). Copper‐free click reactions were conducted to further conjugate the M6P and peptide to the component DNA strands. Denaturing polyacrylamide gel electrophoresis (PAGE) and matrix‐assisted laser desorption/ionization–time‐of‐flight mass spectrometry (MALDI‐TOF MS) were performed to demonstrate the successful modification, as evidenced by slow migration of the bands (Figure S17 and S19, Supporting Information) and the expected m/z values of DBCO‐modified DNA strands (Figure S18 and S20, Supporting Information), M6P‐DNA conjugates (Figure 1b,c), and peptide‐DNA conjugates (Figure 1d,e).

With successful syntheses of M6P and peptide‐modified DNA strands, the LYTAC Plus hydrogel was prepared via nucleic acid self‐assembly (Figure 1a). Specifically, the M6P‐Y‐motif, peptide‐modified DNA linker (Peptide‐linker), and siRNA linker that target ANG‐2 gene were annealed separately and mixed with a 1:1.4:0.1 ratio at room temperature, resulting in LYTAC Plus hydrogel formation through well‐established sticky end hybridization. As a control in our study, similar hydrogel only with LYTAC function (LYTAC gel) was prepared using the same protocol by barely mixing M6P‐Y‐motif and peptide linker with a 1:1.5 ratio. The successful assembly of LYTAC Plus was confirmed using 10% native PAGE, with both the M6P‐Y‐motif and peptide‐modified linker appearing as a single sharp band with slower mobility. As LYTAC Plus had a larger assembled structure, it was almost unable to move into the native gel (Figure 1f). Additionally, SEM analysis revealed the interwoven network of the LYTAC Plus hydrogel (Figure 1g).

Rheological tests were conducted to further confirm the hydrogel formation of LYTAC Plus, in which frequency sweep tests between 0.08 and 16 Hz at a fixed strain of 1% at 37 °C showed a higher shear storage modulus (*G′*) than the shear loss modulus (*G′′*) (Figure 1h). Furthermore, temperature‐ramp rheological tests from 30 to 70 °C at a fixed frequency (1 Hz) and a strain (1%, Figure 1i) revealed that *G′* gradually decreased when the temperature increased. There was an intersection between *G′* and *G′′* ≈67 °C, indicating a gel‐to‐sol transition resulting from the disassociation of complementary base pairing between different DNA strands.^[^ ^16a^ ^]^ The particle size of LYTAC Plus was easily adjusted by changing the mix proportion and concentration of Y‐motif and linkers, with a concentration of 2 µM (Y‐motif:linkers = 1:1.4), yielding nanogels with uniform size distribution (Figure S21, Supporting Information).

The disassembly ability of LYTAC Plus gel was investigated by incubating it with different concentrations of nuclease solutions or cell culture medium. Based on the results of dynamic light scattering (DLS) measurement (Figure S22, Supporting Information), we could observe that within 12 h incubation at 37 °C, the sizes of obtained small gel particles decreased along with the promotion of nuclease concentrations (5‐50 U mL^−1^). LYTAC Plus was degraded into smaller particles in the cell culture medium containing 1% or 5% FBS (Figure S23, Supporting Information). To confirm the functional siRNA release, LYTAC Plus was treated with different concentrations of RNase H at 37 °C for 1 h and analyzed on 10% native PAGE. In the presence of RNase H, LYTAC Plus released siRNA in a concentration‐dependent manner (Figure S24, Supporting Information), consistent with our previous findings.^[^ ^11b^ ^]^

### 2.2 Lysosome Colocalization and Cellular Uptake of LYTAC Plus Gel

**Figure 2:**
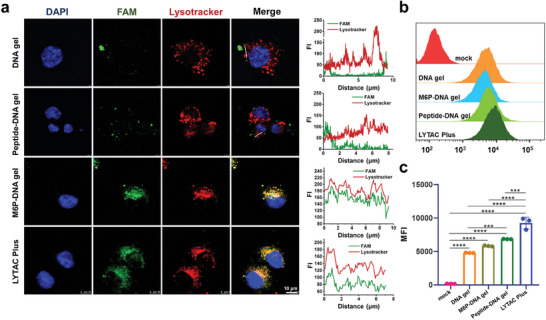
a) Representative CLSM images for lysosome colocalization analysis and fluorescence intensity (FI) profiles of a cross‐section of the CLSM images after incubating HUVECs with different FAM‐labeled materials (3 µM FAM) for 4 h. Scale bar: 10 µm. b,c) Flow cytometry results of HUVECs after 6 h of incubation with FAM‐labeled different materials. The statistical significance was determined using the analysis of variance and a two‐tailed Student's t‐test. Statistical significance was noted as follows: *p < 0.05; **p < 0.01; ***p < 0.001; ****p < 0.0001.

Next, we investigated whether M6P could confer an active targeting ability to our prepared LYTAC Plus toward lysosomes. Lysosome colocalization was assessed to determine whether M6P molecules could enhance the transportation of the LYTAC Plus gel to lysosomes. As shown in **Figure** **2**a; Figure S25 (Supporting Information), DNA hydrogel only with M6P modification (M6P‐DNA gel) and LYTAC Plus gel (green) clearly colocalized with lysosomes (red) in human umbilical vein endothelial cells (HUVECs) after 4 h of incubation, based on confocal laser scanning microscopy (CLSM) imaging. By contrast, DNA gels without M6P modification (DNA gel) did not exhibit this effect, confirming M6P‐mediated lysosome trafficking via M6P‐M6PR interaction in endothelial cells (Figure S26, Supporting Information). We also observed that LYTAC Plus gel took longer time to colocalize with lysosomes compared to the reported LYTAC materials in the literature.^[^ ^5^, ^8^ ^]^ This may be due to the fact that as a nucleic acid hydrogel material, LYTAC Plus needs to be enzymatically degraded to a suitable size for cellular uptake before being transported to the lysosomes by M6PR.

Further, we examined the cell internalization ability of the LYTAC Plus gel. The uptake efficiencies by HUVECs were compared among DNA gels with and without M6P modifications, only with peptide modification (peptide‐DNA gel), and LYTAC Plus gel after 6 h of incubation. The flow cytometry results demonstrated that M6P‐DNA gel or peptide‐DNA gel had a slightly better cellular uptake than DNA gel without any modifications (Figure 2b,c; Figure S27, Supporting Information). Meanwhile, the LYTAC Plus gel exhibited the best cellular uptake efficiency among all samples, probably owing to its synchronous binding with M6PR and VEGFR‐2. These findings suggest that the LYTAC Plus gel has the ability to traffic toward lysosomes and demonstrate efficient cellular uptake.

### 2.3 Protein Degradation Capability of LYTAC Gel

After successfully preparing LYTAC Plus and confirming its efficient cellular uptake, we further investigated its protein degradation capability from an in vitro perspective, using n‐AMD as the disease model. Neovascularization is a key pathological feature of retinal diseases.^[^ ^25^ ^]^ VEGF overexpression has been linked to the etiology of neovascular eye disease.^[^ ^26^ ^]^ The physiological release of VEGF is crucial for maintaining vascular integrity, but excessive amounts can cause aberrant vascular proliferation.^[^ ^27^ ^]^ Anti‐VEGF biologic medicines have gained approval in recent years, shifting the clinical situation from one in which there was essentially no treatment for these disorders to one with routine treatment for n‐AMD. Monoclonal antibodies are created in the lab and used in the clinic in accordance with the pathophysiology to successfully treat n‐AMD. Anti‐VEGF drugs have evolved over numerous generations, starting with the original Macugen and oligonucleotides of VEGF‐A_165_ monomers and progressing to monoclonal antibodies Avastin and Lucentis and, later, antibody fusion proteins Aflibercept and Conbercept, which bind to more VEGF targets with considerably more potent effects.^[^ ^28^ ^]^ The interaction between VEGF‐A and VEGFRs plays a crucial role in neovascularization development.^[^ ^29^ ^]^ VEGF‐A has multiple isoforms, and VEGF_165_ is the most important subtype in the human body, acting as an effective agonist of VEGFR‐1 and VEGFR‐2.^[^ ^18a^ ^]^ As such, VEGF_165_ is often used as a stimulating factor to study endothelial cell angiogenesis.^[^ ^30^ ^]^ In this study, VEGF_165_ was also utilized to stimulate HUVECs and HRMECs to simulate the pathological environment found in patients with n‐AMD.

**Figure 3:**
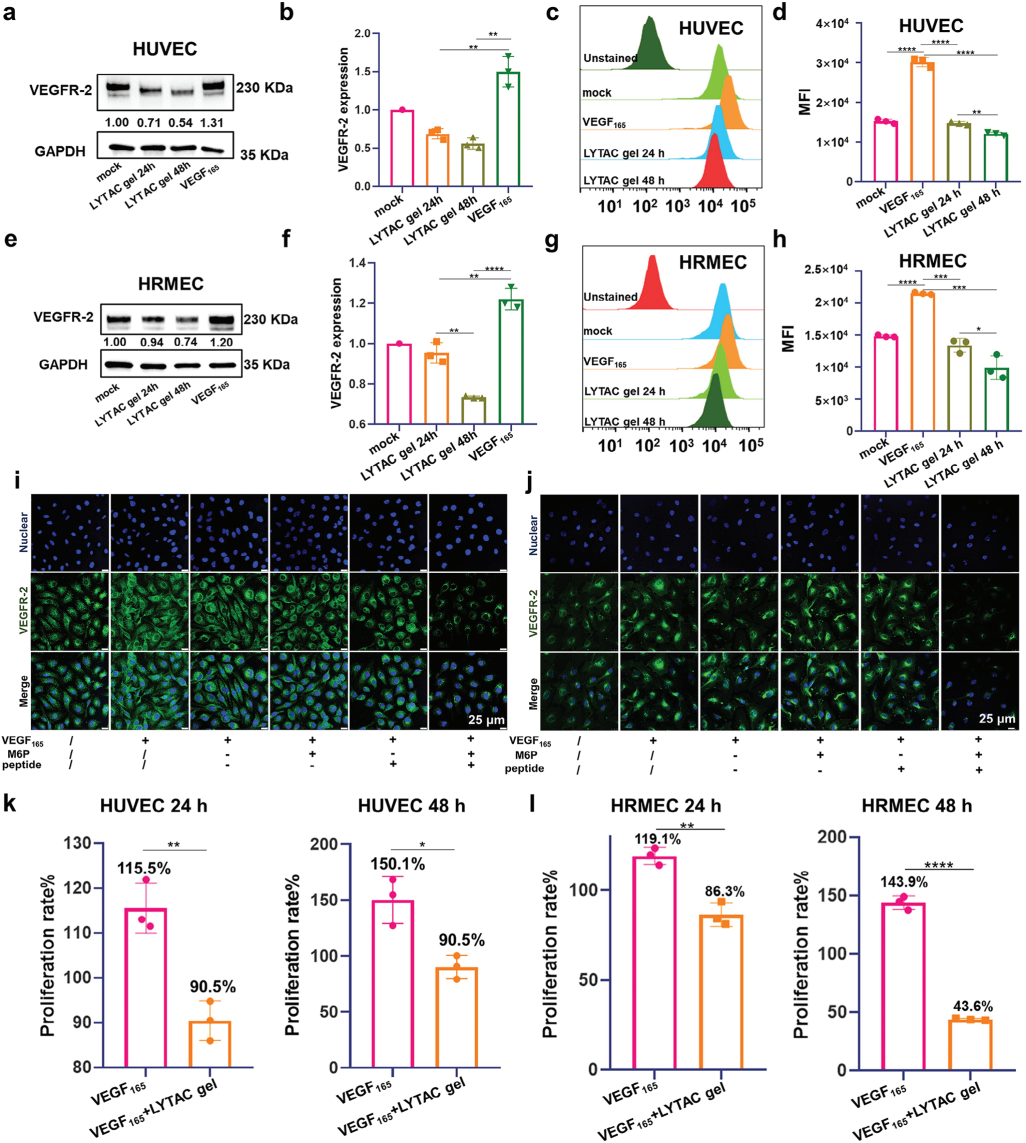
Protein degradation and anti‐proliferation capability of the LYTAC gel. Western blot analysis of total VEGFR‐2 levels after a,b) HUVECs and e,f) HRMECs were treated with 2 µM LYTAC gel for 24 and 48 h under the stimulation of VEGF_165_ (50 ng mL^−1^). Degradation of VEGFR‐2 in c,d) HUVECs and g,h) HRMECs as determined by flow cytometry after 24 h and 48 h treatment of 2 µM LYTAC gel under the stimulation of VEGF_165_ (50 ng mL^−1^). Visualization of VEGFR‐2 degradation in i) HUVECs or j) HRMECs by CLSM after treatment with 2 µM different samples under the stimulation of VEGF_165_ (50 ng mL^−1^). Scale bar: 25 µm. CCK‐8 assay‐based cell proliferation tests of (k) HUVECs and (l) HRMECs treated with 2 µM LYTAC gel for 24 h and 48 h compared with cells stimulated with only VEGF_165_ (50 ng/mL). The statistical significance was determined using the analysis of variance and a two‐tailed Student's t‐test. Statistical significance was noted as follows: *p < 0.05; **p < 0.01; ***p < 0.001; ****p < 0.0001.

To demonstrate the protein degradation capability of our obtained LYTAC hydrogels, the abundance of its targeting membrane protein, VEGFR‐2, on the cell surface was thoroughly investigated and monitored using various methods. As ANG‐2 siRNA in LYTAC Plus gel only accounts for 1/15 of the total linkers, to avoid the potential interference of targeting protein expression by ANG‐2 siRNA, LYTAC gel was simply employed as a substitute to validate their protein degradation capabilities. Western blot analysis revealed that the level of VEGFR‐2 in HUVECs decreased by ≈60% at 24 h and 77% at 48 h when treated simultaneously with 50 ng mL^−1^ VEGF_165_ and 2 µM LYTAC gel, compared to cells stimulated with only VEGF_165_ (**Figure** **3a**,b). Flow cytometry experiments also showed that the LYTAC gel significantly reduced the expression of VEGFR‐2 in HUVECs within 24 h and 48 h (Figure 3c,d). Similar results were observed in the HRMEC case, with western blot analysis demonstrating a 26% decrease in VEGFR‐2 levels after 24 h and a 46% decrease after 48 h when treated with VEGF_165_ and LYTAC gel (Figure 3e,f), which was further confirmed by flow cytometry experiments (Figure 3g,h). Immunofluorescence experiments demonstrated that only the material with both M6P and targeting peptide modifications could efficiently reduce the expression of VEGFR‐2 in either HUVEC or HRMEC cells, owing to the presence of the LYTAC function (Figure 3i,j).

Upon degrading the VEGFR, the anti‐angiogenic effect of LYTAC gel on endothelial cells was assessed using CCK‐8 assays and wound healing tests. Compared to the VEGF_165_ stimulated group, LYTAC gel inhibited the proliferation of HUVECs and HRMECs by ≈25.0% and 32.8% within 24 h and by 59.6% and 100.3% within 48 h, respectively, (Figure 3k,l). Moreover, the proliferation rate of HUVECs treated with LYTAC gel was much lower than that of the VEGF_165_ stimulated group and other treated groups that did not possess the LYTAC function (Figure S28, Supporting Information). These results indicate that LYTAC gel can effectively inhibit the proliferation of endothelial cells by VEGFR‐2 degradation.

A wound healing assay was conducted to assess the migration of HUVEC after its co‐incubation with VEGF_165_ and test samples. Cells were seeded into a 6‐well plate, scratched with a 200 µL pipette micro tip, and photographed at 0, 12, 24, and 48 h after treatment. After 48 h, the scratches of the groups stimulated by only VEGF_165_ or DNA gel without any modification healed completely, while the scratches of HUVECs treated with LYTAC gel were still apparent (Figure S29, Supporting Information). This indicates that the LYTAC gel has a favorable anti‐migration capability.

### 2.4 Gene Silencing by LYTAC Plus Hydrogel

However, the efficacy and dosage frequency of the currently available VEGF inhibitors warrant improvement. The fact that when VEGF activity is inhibited, the production of other pro‐angiogenic factors is upregulated, limiting the efficacy of VEGF inhibitors, presents a significant obstacle to single‐target anti‐VEGF therapy.^[^ ^31^ ^]^ The currently licensed VEGF inhibitors also have a short half‐life, necessitating frequent administration, such as once a month for ranibizumab.^[^ ^32^ ^]^ A higher frequency of intraocular injections is likely to make patients uncomfortable, which will decrease adherence to treatment regimens because current VEGF inhibitors are given to patients by intravitreal injection.^[^ ^33^ ^]^ Thus, there is still a significant unmet clinical need for the management of n‐AMD.

Anti‐VEGF drug development for the treatment of eye illnesses is now moving in an essential direction toward dual‐target medications.^[^ ^34^ ^]^ The FDA authorized Roche's bispecific antibody Vabysmo (faricimab‐svoa) on January 28, 2022, which is fascinating to note.^[^ ^22b^ ^]^ Vabysmo is a highly focused double antibody that inhibits both VEGF‐A and ANG‐2.^[^ ^22b^ ^]^ Besides VEGF, ANG‐2 is another crucial angiogenic factor that plays a role in both pathological and healthy angiogenesis. Recent research has revealed a strong correlation between ocular disorders and aberrant ANG‐2 expression.^[^ ^35^ ^]^ Bispecific antibodies against ANG‐2 for eyes have made substantial progress in the treatment of n‐AMD, diabetic macular edema (DME), and other retinal illnesses.^[^ ^36^ ^]^ In reaction to inflammation or hypoxia, ANG‐2 can be secreted in an aberrant manner.^[^ ^37^ ^]^ As an Ang‐1 antagonist, ANG‐2 competitively binds to Tie‐2, inhibiting the vascular stabilizing effect of ANG‐1.^[^ ^38^ ^]^ This causes endothelial cells to become unstable and disconnected from neighboring cells, which causes vascular instability and endothelial activation. In the presence of VEGF, ANG‐2 works in concert to encourage endothelial cells to accept the budding signal of VEGF and other angiogenic inducers, resulting in the continual formation of new blood vessels in tissues.^[^ ^39^ ^]^ Considering the disease characteristics of n‐AMD and the current status of clinical research, we chose to incorporate ANG‐2 as the second therapeutic target in the LYTAC Plus gel.

**Figure 4:**
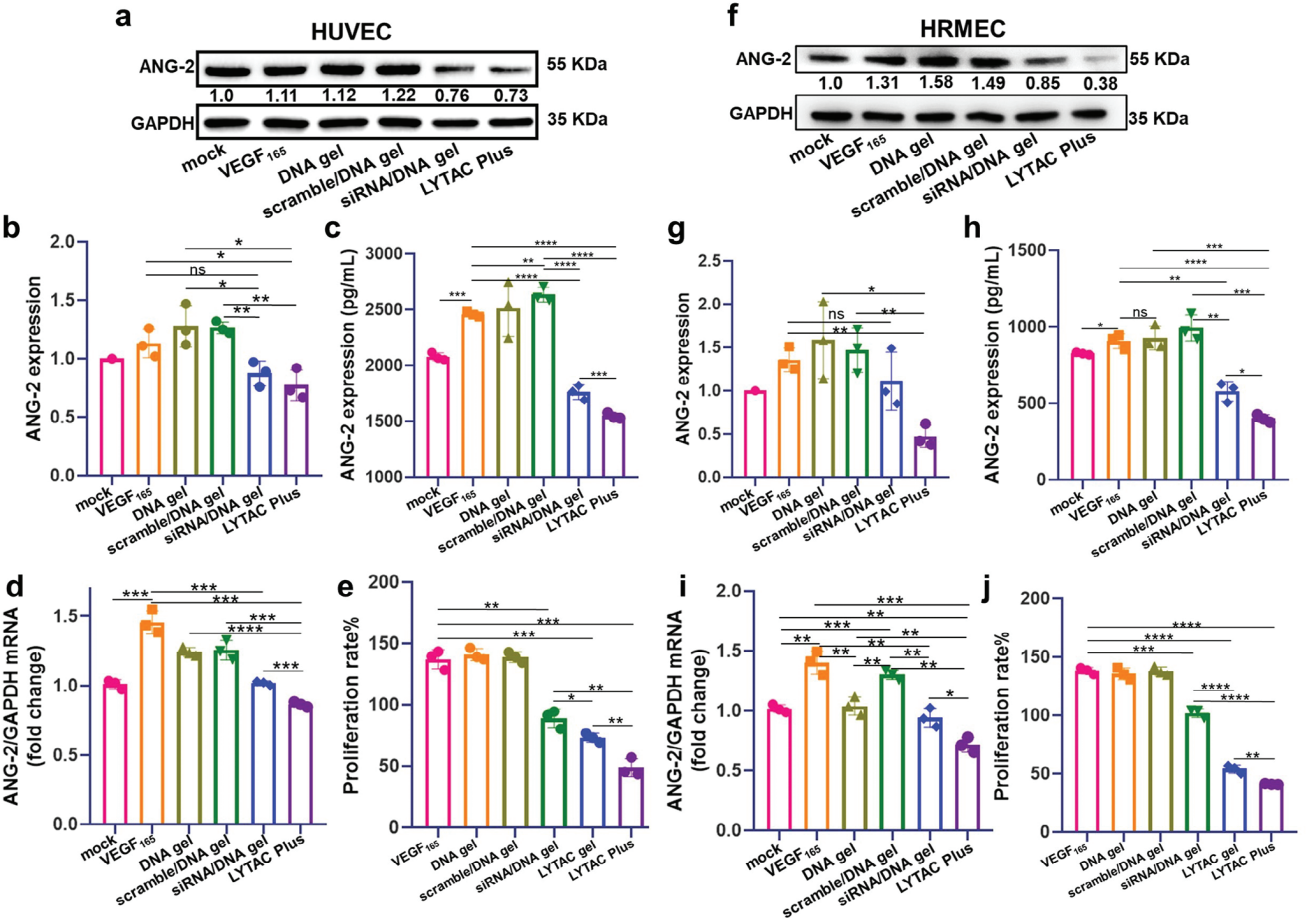
Gene silencing and anti‐proliferation effects of LYTAC Plus gel in vitro. Intracellular ANG‐2 protein expression of a,b) HUVECs and f,g) HRMECs after transfection with DNA gel, DNA gel with scramble siRNA (scramble/DNA gel), DNA gel with ANG‐2 siRNA (siRNA/DNA gel), and LYTAC Plus gel (2 µM Y‐motif, 200 nM siRNA) for 6 h and incubation for 48 h under the treatment of VEGF_165_ (50 ng mL^−1^). c,h) Secreted ANG‐2 protein levels of HUVECs and HRMECs treated with different formulations for 48 h under the stimulation of VEGF_165_. ANG‐2 mRNA expression in d) HUVECs and i) HRMECs after treatment with different formulations for 48 h. CCK‐8 assay‐based cell proliferation tests of e) HUVECs and j) HRMECs treated with (2 µM Y‐motif, 200 nM siRNA) different formulations for 48 h and VEGF_165_ compared with cells that were stimulated with only VEGF_165_ (50 ng mL^−1^). The statistical significance was determined using the analysis of variance and a two‐tailed Student's t‐test. Statistical significance was noted as follows: *p < 0.05; **p < 0.01; ***p < 0.001; ****p < 0.0001.

After validating the protein degradation capacity of LYTAC gel, the gene silencing ability of LYTAC Plus gel was investigated in detail using siRNA to downregulate ANG‐2, another important driving force in n‐AMD development. As such, HUVECs and HRMECs were separately transfected with DNA gel without any modification, DNA gel loaded with ANG‐2 scramble siRNA (scramble/DNA gel), DNA gel loaded with ANG‐2 siRNA (siRNA/DNA gel), and LYTAC Plus gel for 6 h with VEGF_165_ stimulation and incubated for 48 h. The expression of the ANG‐2 protein was then examined by western blot and ELISA kit. It was observed that both the intracellular and secreted ANG‐2 proteins of HUVECs (**Figure** **4**a–c) and HRMECs (Figure 4f–h) were upregulated by VEGF_165_ stimulation. Although both the siRNA/DNA gel and LYTAC Plus could significantly decrease ANG‐2 protein expression, LYTAC Plus exhibited better performance.

Further, quantitative real‐time polymerase chain reaction (qRT‐PCR) analysis revealed that ANG‐2 mRNA expression was significantly increased in both HUVECs (≈1.45) and HRMECs (≈1.4) by VEGF_165_ stimulation (Figure 4d,i) compared to mock (normalized as 1.0). After the treatments, the siRNA/DNA gel and the LYTAC Plus gel showed a distinct downregulation of ANG‐2 mRNA expression in both HUVEC (≈1.02 and 0.86) and HRMEC cells (≈0.94 and 0.65). The remarkable outcome with the LYTAC Plus gel can be attributed to the LYTAC‐based VEGFR‐2 degradation, which further suppresses the expression of ANG‐2 in endothelial cells. By contrast, DNA gel without any modification or with scramble RNA linkers (scramble/DNA gel) did not significantly knockdown ANG‐2 mRNA expression. Similarly, the anti‐proliferation capability of the LYTAC Plus gel was also investigated. After 48 h incubation with tested samples in the presence of VEGF_165_ stimulation, the proliferation rates on HUVECs and HRMECs were quantified by a CCK‐8 kit (Figure 4e,j), in which DNA gel and scramble/DNA gel did not inhibit the proliferation of HUVECs or HRMECs. As expected, the siRNA/DNA gel, LYTAC gel, and LYTAC Plus were able to suppress endothelial cell proliferation, among which the LYTAC Plus gel exhibited the best anti‐proliferation effect.

The results of Ki67 immunofluorescence staining for HUVECs and HRMECs are demonstrated in Figure S31 and S32 (Supporting Information). Following stimulation with VEGF_165_, the proportion of Ki67‐positive cells greatly increased, indicating the enhanced cell proliferation. However, after treatment with LYTAC or LYTAC Plus gel, the proportion of Ki67‐positive cells in both HUVEC and HRMEC significantly decreased along with the incubation time. Further, with the same incubation time, the proportion of Ki67‐positive cells in cells treated with LYTAC Plus was noticeably lower than that of LYTAC‐treated group. These results indicate that LYTAC Plus gel not only exerts an ideal gene silencing effect but also has a better anti‐proliferation effect than LYTAC gel, based on its dual‐function design.

### 2.5 The Safety of LYTAC Plus Gel in an Ocular Application

**Figure 5:**
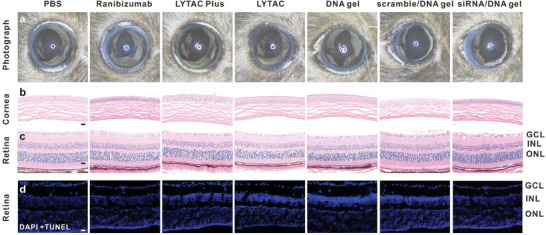
Local toxicity of the designed pharmaceutical regimen. Representative images of the anterior segment a) of treated mice taken 7 days after the model was constructed. Mice (N = 3) from each experimental group's cornea b) and retina c) stained histologically. Scale bar: 20 µm. Mice (N = 3) from each experimental group were stained using TUNEL (red) for apoptotic cells and DAPI (blue) for the nucleus of the retina d). GCL: ganglion cell layer, INL: inner nuclear layer, ONL: outer nuclear layer. Scale bar: 25 µm. N: number of eyeballs.

Prior to examining the effectiveness of treatment in animals, the in vivo biocompatibility of LYTAC and LYTAC Plus was assessed. To this end, the oculars of normal animals were injected with PBS, ranibizumab, LYTAC Plus, LYTAC, DNA gel, scramble/DNA gel, and siRNA/DNA gel, followed by slit‐lamp examinations after one week. The results indicated that all groups remained generally healthy, with clear corneas and the lower iris field unobtrusive under the slit‐lamp microscope (**Figure** **5**a). Moreover, H&E staining of tissue sections from the eyes revealed that the corneal and retinal structure layers were intact and free of pathological abnormalities (Figure 5b,c). TUNEL staining of the retinal slices did not reveal any red fluorescence, indicating the absence of cell apoptosis (Figure 5d). We employed TUNEL labeling and immunofluorescence staining of specific markers for both RPE65 and photoreceptor cells to better demonstrate whether the posterior region of the eye's structure was destroyed. As shown in Figure S33 (Supporting Information), sample injections into mouse vitreous cavities in each group did not cause the apoptosis of both RPE65 and photoreceptor cells. In contrast, a positive control for the TUNEL assay is shown in Figure S34 (Supporting Information), demonstrating a substantial apoptosis of the retina cells. Lastly, H&E staining of the heart, liver, spleen, lungs, and kidneys of the mice showed no significant histopathological abnormalities or lesions (Figure S35, Supporting Information). All these results demonstrated the great biocompatibility and biosafety of both LYTAC gel and LYTAC Plus gel.

### 2.6 Therapeutic Effect of LYTAC Plus on a Mouse Model of Laser‐Induced CNV

**Figure 6:**
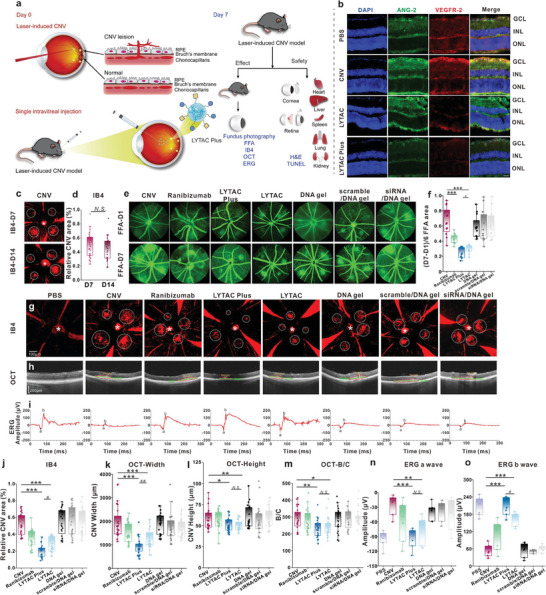
In vivo effectiveness in a mouse model of laser‐induced CNV. a) Experimental timeline and schematics. Retinal laser‐induced photocoagulation (4 spots per eye) and single intravitreal injection were performed on day 0. The yellow dot above (black dotted circle) represents the process of CNV, and the normal area is shown below (black line circle). LYTAC Plus was injected into the vitreous cavity by a microliter syringe. On day 7, fundus photography, FFA, IB4, OCT, and ERG were conducted to evaluate the efficacy of the prescribed medication regimen. H&E and TUNEL staining were used to assess the safety of the drugs. b) Immunofluorescence staining of CNV mouse retinas of ANG‐2 and VEGFR‐2 after treatment with LYTAC or LYTAC Plus for 7 days. GCL: ganglion cell layer, INL: inner nuclear layer, ONL: outer nuclear layer. Scale bar: 50 µm. c,d) Representative IB4 staining graphs and statistical analysis on day 7 and day 14. e,f) Representative FFA images and statistical analysis of the lesion resolution rate in the same mouse before and after the treatment. Based on FFA photos from the same mouse, the rate of lesion resolution is expressed as (the area of laser spots in day 7‐ the area of laser spots in day 1)/6. g–i) Images obtained on day 7 of laser‐induced CNV mice treated with PBS, ranibizumab, LYTAC Plus, LYTAC, DNA gel, scramble/DNA gel and siRNA/DNA gel. The position of the CNV area (white dotted circle) and optic disc (asterisk) is clearly depicted in the fluorescence images of choroid flat mounts (g) stained with IB4 (red). Scale bar: 100 µm. The largest horizontal diameter of a CNV served as the definition of CNV width. The distance between the highest point of the CNV and Bruch's membrane was used to calculate CNV height (h). Scale bar: 200 µm. In step 5, with the stimulus applied at 10 cd × s m^−2^, scotopic (dark‐adapted) ERG responses (i) demonstrated exemplary a‐wave and b‐wave traces in the eyes from the diseased and therapy groups. j–o) Quantitative analysis of relative CNV areas (j), CNV widths (k), heights (l) and B/C ratios (m) on OCT, ERG a‐waves (n), and b‐waves (o) 7 days later after treatment with all groups. The two‐sample T test is used to determine the significance of the data, which are reported as means ± SEM of different studies. The difference among multiple groups was evaluated using a one‐way analysis of variance (ANOVA) with Bonferroni's post hoc test. CNV group compared to LYTAC Plus and LYTAC group: *p < 0.05, **p < 0.01, ***p < 0.001. The LYTAC Plus group compared to the LYTAC group: ^#^ *p* < 0.05, ^##^ *p* < 0.01, ^###^ *p* < 0.001. No significance, or N.S.

Following an established procedure, we used laser photocoagulation to produce an experimental n‐AMD model in mice. Due to increased microvascular permeability in n‐AMD, the development of new blood vessels resembles the original blood vessels branching off, which results in bleeding (**Figure** **6**a). Immunofluorescence experiments were conducted to investigate the expression levels of the ANG‐2 and VEGFR‐2 proteins in the mouse retina. As shown in Figure 6b, CNV mice had considerably higher levels of ANG‐2 and VEGFR‐2 expression in their retinas than did mice in the PBS group. After treatment with LYTAC or LYTAC Plus, the expression levels of ANG‐2 and VEGFR‐2 were greatly reduced compared to the CNV group. Further, the expression level of the targeted proteins in the LYTAC Plus group was lower than that in the LYTAC group. Following our initial statistical analysis on day 7 and day 14, we found no statistically difference between the two treated groups (Figure 6c,d). Further, we evaluated the therapeutic effects of different samples, which was based on the FFA images using (the area of laser spots in day 7‐ the area of laser spots in day 1)/6 to determine the average rate of lesion resolution from the same mouse before and after treatment. As shown in Figure 6e,f, the results indicated that groups treated with LYTAC Plus had the smallest leakage fluorescence area (n = 12). The CNV lesion area was then quantified using red fluorescence of isolectin IB4 (a vascular marker) staining on choroid tiles from mice that had also received fundus fluorescein angiography (FFA) and optical coherence tomography (OCT) treatment. Both the LYTAC Plus (n = 24, n: number of laser spots) group and LYTAC treatment group (n = 24) significantly reduced the CNV regions compared to the laser‐induced CNV (n = 24) mice, and the LYTAC Plus group showed more obvious suppression than the LYTAC treated group (Figure 6g,j). Meanwhile, OCT scan images showed that CNV width was significantly reduced in groups treated with LYTAC Plus and LYTAC‐treated compared to laser‐induced CNV mice (Figure 6h,k), in which LTAC Plus gel was even more capable of minimizing CNV width. Similar trend could also be confirmed by the analysis of ratios (B/C) of lesion thickness (B) from the bottom of the pigmented choroid layer to the top of the neovascularization membrane to the intact pigmented choroid thickness (C) adjacent to the lesion, which could minimize the inaccuracy caused by imaging at different angles during the measurements (Figure 6m). According to the OCT findings, LYTAC Plus had a superior ability than LYTAC to reduce the breadth of CNV, although there was no significant difference between the two groups regarding CNV height (Figure 6l). However, LYTAC Plus had the greatest reduction in the overall CNV area. The quantification of the CNV lesion area by IB4 staining showed that the reduction in overall CNV area was most pronounced in the LYTAC Plus group.

n‐AMD, which can cause a rapid loss of central vision due to aberrant blood vessel growth into the macula and scarring from blood or fluid leakage, is typically described as central vision loss characterized by progressive macular degeneration.^[^ ^40^ ^]^ In the development process of AMD, the retinal pigment epithelium (RPE) cells are damaged first, resulting in morphological and functional changes and loss of RPE cells, and eventually the Bruch membrane, RPE layer, photoreceptor cell layer (cone and rod cells), and other neuron cell layers (horizontal cells, bipolar cells, etc.) are gradually damaged. In the present study, we investigated the functionality of these cells using electroretinograms (ERGs) (Figure 6i). The ERG a‐wave mainly comes from the receptor potential of photoreceptor cells, and the ERG b‐wave mainly reflects the function from the inner plexus layer to the ganglion cell layer (including bipolar cells, Müller cells and ganglion cells, etc.). Quantitative analysis of scotopic ERG responses revealed that the LYTAC Plus (N = 6, N: number of eyeballs) and LYTAC‐treated (N = 6) groups both significantly improved a‐wave signals compared to the CNV (N = 6) eyes without treatment, but with no statistic differences between former two treatment groups (Figure 6n). In contrast to a‐wave, b‐wave signals in the LYTAC plus group showed a greater improvement compared to that of LYTAC group, both of which were significant stronger than that of CNV group, indicating that LYTAC Plus gel could better restore the inner retina's electrophysiological function (Figure 6o). The results highlight that LYTAC Plus‐driven degradation can exert more profound effects on electrophysiological function. These results indicate that ANG‐2 protein knockdown and effective VEGFR‐2 degradation could prevent the development of neovascularization in the mouse retina. Additionally, these findings suggest that LYTAC Plus, which can act on two targets, is more therapeutically effective than LYTAC, which only targets one pathway.

### 2.7 Biodistribution of LYTAC Plus Gel In Vivo

Finally, we investigated the metabolic disassembly process of LYTAC Plus in mice. Intravitreal injection of Cy5‐labeled LYTAC Plus gel in mice was used to investigate its distribution in major organs within 7 days after administration. On days 1, 3, and 7, the organs, blood, and ocular tissues of mice were collected, and the Cy5 fluorescence signals were recorded. No Cy5 fluorescence signals were detected in the heart, liver, spleen, lung, or kidney of the mice within 7 days (Figure S36a, Supporting Information). At the same time, Cy5 content detected in the blood on days 1, 3, and 7 did not exceed 0.1% of the Cy5‐labeled sample injected into the vitreous (Figure S36b, Supporting Information). These results indicate that LYTAC Plus gel injected into the vitreous does not enter the main organs or blood circulation of mice. Moreover, distribution of the LYTAC Plus gel in different parts of the eyes showed that the injecting gel was mainly concentrated in the retina, with a small amount distributed in the cornea, conjunctiva, choroid, lens, and optic nerve within 7 days after administration (Figure S36c,d, Supporting Information).

## 3 Conclusion

In summary, we proposed the concept of LYTAC Plus, and verified the feasibility of LYTAC Plus through the delivery vector of nucleic acid hydrogel in this study. We successfully synthesized DNA hydrogel‐based LYTAC Plus with both the membrane protein degradation function of LYTACs and the gene silencing ability of siRNA. Using n‐AMD as the disease model, we proved that LYTAC Plus not only degraded the membrane protein VEGFR‐2 but also downregulated ANG‐2 expression. Moreover, compared with LYTAC gel, LYTAC Plus presented a better anti‐angiogenesis ability and a better therapeutic effect both in vitro and in vivo. LYTAC Plus confers LYTACs new functions and broadens its application range. According to the different mechanisms of disease development, LYTAC Plus can achieve an ideal therapeutic effect by changing the targeted POIs or siRNA sequences of genes that need to be downregulated. Although LYTAC Plus exhibits many advantages, there are still many problems worthy of further studies in future works, such as the specific pathway of LYTAC Plus in cells and lysosome escape. A better understanding of the mechanism of LYTAC Plus can foster the application of this technology in the treatment of clinical diseases.

## 4 Experimental Section

### Synthesis of M6P‐Modified and VEGF Peptide‐Modified DNA

DBCO‐NHS ester (50 equiv.) was added to the amino‐modified DNA solution (14.5 OD mL^−1^, 20% H_2_O/DMSO), and reacted for 24 h with shaking at 25 °C. Th excess DBCO‐NHS ester was then removed by extraction with ethyl acetate 6–8 times, after which the DBCO‐modified DNAs (DBCO‐Y‐a, DBCO‐Y‐b, DBCO‐Y‐c, and DBCO‐linker‐1) were acquired by removing the water under vacuum and stored at −20 °C for further use. As for the M6P‐modified DNAs, M6P‐N_3_ (5 equiv.) was added to DBCO‐Y‐a, DBCO‐Y‐b, and DBCO‐Y‐c (200 µM, DMSO) and reacted for 48 h with shaking at 50 °C.^[^ ^41^ ^]^ Then the solution was dialyzed to remove the excess M6P‐N_3_ and DMSO, and M6P‐modified DNAs (M6P‐Y‐a, M6P‐Y‐b, and M6P‐Y‐c) were stored at −20 °C for further use. The VEGF peptide‐modified DNA was prepared using the same method. Denaturing polyacrylamide gel, which contains 20% acrylamide (19:1, acrylamide/bisacrylamide) with 8 M urea, was used to characterize the different samples in 1 × TBE buffer (89 mM Tris, 89 mM boric acid, and 2 mM EDTA). Gels were imaged under the BioRad imaging system.

### Preparation of LYTAC Plus

To construct the M6P‐containing Y‐motif, an equimolar amount of three M6P‐modified DNAs were mixed in 1 × TAE/Mg^2+^ buffer (40 mM Tris, 2 mM EDTA·Na·H_2_O, 20 mM acetic acid, 12.5 mM (CH_3_COO)_2_Mg·4H_2_O, pH = 7.4, adjusted by acetic acid), which was slowly cooled down from 90 °C to 25 °C, and the DNA linkers with peptide or siRNAs were annealed by cooling from 75 °C to 25 °C in 1 × TAE/Mg^2+^ buffer. The LYTAC Plus was prepared with a Y‐motif and linker ratio of 1:1.5 at room temperature for 30 min,^[^ ^16a^ ^]^ and 10% native polyacrylamide gel electrophoresis and 1% agarose gel electrophoresis were used to characterize the samples in 1 × TAE/Mg^2+^ buffer. The morphology of the LYTAC Plus was characterized by a transmission electron microscope (TEM, JEM‐2010/INCA OXFORD) and field‐emission scanning electron microscopy (SEM, Sirion 200, FEI, USA). The size of the LYTAC Plus nanogel was measured by dynamic light scattering on a Zetasizer Nano ZS instrument (Marvern Instruments, Malvern). The scattering angle was kept at 173°, and the wavelength of laser light was 633 nm during the whole experiment. FAM‐modified materials were constructed in the same way as described above by altering linker‐2 DNA with FAM‐linker‐2.

### RNase H‐Mediated siRNA Release from LYTAC Plus

The LYTAC Plus was incubated with different concentrations of RNase H (0, 10, 20, 50, and 100 U mL^−1^) for 1 h at 37 °C. Then, 10% native PAGE electrophoresis was conducted to characterize the release of siRNA segments.^[^ ^11b^ ^]^

### Cell Culture

Primary human umbilical vein endothelial cells (HUVECs) (ScienCell, Carlsbad, CA, USA; Cat.#: 8000) were cultured in endothelial cell growth media (Endothelial Cell Growth Medium‐2 BulletKit, Lonza, Cat.#: CC‐3162) and primary human retinal microvascular endothelial cells (HRMECs) (Cell Systems, Kirkland, WA, USA, Cat. #ACBRI 181) were cultured in microvascular endothelial cell growth medium (Microvascular Endothelial Cell Growth Medium‐2 BulletKit, Lonza, Cat.#: CC‐3202) at 37 °C in a humidified atmosphere containing 5% CO_2_.

### Lysosomal Colocalization Experiment In Vitro

CLSM was used to visualize the lysosomal colocalization of different samples. HUVECs (2 × 10^4^) and HRMECs were seeded into a 12‐well plate with a cover slip at the bottom of each well and cultured in EGM‐2 overnight. After discarding the media and washing twice with PBS solution, the cells were treated with different FAM‐labeled samples (2 µM in opti‐MEM, DNA gel, M6P‐DNA gel, peptide‐DNA gel, LYTAC Plus) at 37 °C for 2 or 4 h. The cells were then washed with PBS solution twice and incubated with LysoTracker Red (C1046, Beyotime) at 37 °C according to the instructions. After being labeled with LysoTracker Red, the cells were washed twice and fixed with 4% formaldehyde for 20 min at room temperature. The cell nucleus was stained with DAPI for 10 min, followed by removing the DAPI solution and rinsed with PBS solution thrice. The cover slides were mounted under dark conditions and visualized using CLSM (Leica TCS SP8 STED 3X).

### In Vitro Cell Proliferation Experiment of LYTAC Gel

The CCK‐8 kit (C0038, Beyotime) was utilized to investigate the antiproliferation effect of LYTAC gel against HUVECs and HRMECs stimulated by VEGF_165_ (P5561, Beyotime). Cells were seeded into 96‐well plates at a density of 5 × 10^3^ cells per well and cultured in EGM‐2 overnight. After the cells were starved in 0.5% EBM‐2 for 6 h, VEGF_165_ (50 ng mL^−1^) mixed with LYTAC gel (2 µM) was added into wells and incubated in culture media with 2% FBS for 24 and 48 h. Then, 10 µL CCK‐8 solution was added to each well and incubated for another 4 h. After incubation, the absorbance value of the wells at 450 ng mL^−1^ was directedly measured by BioTek.

To assess the anti‐proliferation effect of the different samples, cells were seeded into 96‐well plates at a density of 5 × 10^3^ cells per well, which were also cultured in EGM‐2 overnight and starved in 0.5% EBM‐2 for 6 h. The cells were then treated with different samples for 48 h. After incubation, the culture media was refreshed and 10 µL CCK‐8 solution was added to the wells. After incubation for another 4 h, the absorbance value of the wells at 450 ng mL^−1^ was measured by BioTek. The cell proliferation rate was defined as: (OD_sample_–OD_blank_)/(OD_positive_–OD_blank_) × 100%. OD_sample_ represents the absorbance values measured using a microplate reader after incubation with the sample. OD_blank_ represents the background absorbance values obtained with EGM‐2 medium only, without cells. OD_positive_ represents the absorbance values after cell attachment, cultured solely in an EGM‐2 medium without the addition of VEGF_165_ or samples.

### Wound Healing Assay of LYTAC Gel

HUVECs were seeded into a 6‐well plate at a density of 1 × 10^5^ cells per well and cultured in EGM‐2 overnight. A wound was created by scratching the bottom of the well using a 200 µL peptide tip. After the cells were washed with PBS 2 times, different samples mixed with VEGF_165_ (50 ng mL^−1^) were added to the wells and cultured in culture media with 2% FBS for 12, 24, and 48 h. The cell migration rate was calculated using Image J, based on the wounded area photographed by an inverted microscope.

### Protein Degradation Analysis using Western Blot

HUVECs (1 × 10^5^) and HRMECs (1 × 10^5^) seeded into a 6‐well plate were starved for 6 h in 0.5% FBS EBM‐2 and treated with the mixture of VEGF_165_ (50 ng mL^−1^) and LYTAC gel (2 µM) for 24 and 48 h. The cells were then lysed with RIPA buffer supplemented with protease and phosphatase inhibitor (P1045, Beyotime) to acquire the cellular protein, which was quantified by using the BCA kit (P0010, Beyotime). The samples were mixed with loading buffer, denatured at 100 °C for 5 min, and separated on 4–12% SDS‐PAGE gels (ET12412, ACE Biotechnology). Proteins were then transferred onto polyvinylidene difluoride membranes and blocked with 2% skimmed milk in TBST solution for 1 h at room temperature. After being washed with TBST thrice, the membranes were incubated with primary VEGFR‐2 (1:1000, 2479, CST) or GAPDH (1:20 000, 60004‐1‐Ig, Proteintech) antibodies individually at 4 °C for 24 h. The membranes were then washed with TBST thrice and incubated with secondary antibodies (1:5000, Proteintech) at room temperature for 2 h. Then membranes were further washed with TBST thrice and visualized by ECL detection.

### Protein Degradation Analysis by Confocal Microscopy

HUVECs (2 × 10^4^) and HRMECs (2 × 10^4^) were seeded into a 12‐well plate with a cover slip at the bottom of each well and cultured in EGM‐2 overnight. After being starved for 6 h in 0.5% FBS EBM‐2, the cells were treated with VEGF_165_ (50 ng mL^−1^) and different samples for 48 h. The cells were then washed with PBS thrice and fixed with 4% paraformaldehyde for 30 min at room temperature. The cells were further washed, blocked with 5% BSA in PBS for 30 min at room temperature, and incubated with primary antibody (67407‐1‐Ig, Proteintech) at 4 °C for 12 h. The cells were then washed with PBS and incubated with FITC‐labeled secondary antibody (abs20012, Absin) and DAPI. Finally, the cover slides were mounted under dark conditions and visualized using CLSM.

### Protein Degradation Analysis by Flow Cytometry

HUVECs (1 × 10^5^) and HRMECs (1 × 10^5^) which were seeded into a 6‐well plate were starved for 6 h in 0.5% FBS EBM‐2 and treated with the mixture of VEGF_165_ (50 ng mL^−1^) and LYTAC gel (2 µM) for 24 and 48 h. Afterward, adherent cells were trypsinized, washed thrice with FBS stain solution, and fixed with 4% paraformaldehyde for 20 min at room temperature. Then, cells were blocked with 5% BSA in FBS stain buffer solution for 30 min at room temperature and incubated with primary antibody at 4 °C for 30 min (67407‐1‐Ig, Proteintech). Next, the cells were washed with FBS stain solution and incubated with FITC‐labeled secondary antibody (abs20012, Absin). Finally, cells were washed and resuspended in 200 µL stain buffer for the flow cytometry test (LSRFortessa, Becton Dickinson Immunocytometry Systems).

### In Vitro ANG‐2 Downregulation by LYTAC Plus

HUVECs and HRMECs were seeded in 6‐well plates with a density of 1 × 10^5^ cells per well and cultured overnight. After being starved with 0.5% FBS EBM‐2 for 6 h, the cells were treated with VEGF_165_ (50 ng mL^−1^) and transfected with different samples (2 µM, siRNA 200 nM) for 6 h in Opti‐MEM. Then, EBM‐2 with 5% FBS was added into the well and cultured until 48 h. The culture medium was collected for ELISA analysis according to the manufacturer's protocol (EK1215, Lianke Biotech), and cells were lysed for western blot experiments to quantify the expression level of ANG‐2 protein (ANG‐2 primary antibody, ab155106, Abcam).

### Quantitative Real‐Time PCR (qRT‐PCR) Analysis

Total RNA from HUVECs and HRMECs was acquired using RNAeasy Plus Animal RNA Extraction Kit (R0032, Beyotime) according to the manufacturer's protocol and quantified by nanodrop. Reverse transcription of RNA into cDNA was conducted according to the instructions of PrimeScript RT Master Mix (RR036A, TaKaRa). Then qRT‐PCR analysis was performed with TB Green Premix Ex Taq II (RR820A, TaKaRa) by using LightCycler 96 Instrument (Roche).

### In Vitro Anti‐Proliferation Effect by LYTAC Plus

Cells (5 × 10^3^) were plated in a 96‐well culture plate overnight and starved for 6 h before the transfection of siRNA‐bearing materials. Next, the cells were stimulated with VEGF_165_ (50 ng mL^−1^) and transfected with different samples (2 µM, siRNA 200 nM) for 6 h in Opti‐MEM. Then, 5% FBS EBM‐2 culture medium was added into each well and incubated for 48 h. Afterward, 10 µL CCK‐8 solution was added to each well and incubated for another 4 h. After incubation, the absorbance value of the wells was directedly measured at 450 ng mL^−1^.

HUVECs (5 × 10^4^ cell mL^−1^) or HRMECs (5 × 10^4^ cell mL^−1^) were seeded into a 12‐well plate with a cover slip at the bottom of each well and cultured in EGM‐2 overnight. After being starved for 6 h in 0.5% FBS EBM‐2, the cells were treated with VEGF_165_ (50 ng mL^−1^) and different samples for 24 or 48 h. After washing with PBS solution thrice, the cells were fixed and permeabilized with ice‐cold methanol for 15 min at room temperature and washed with PBS. Next, cells were blocked with 5% goat serum/0.3% Triton‐100 in PBS at room temperature for 1 h and incubated overnight with Ki67 antibody (1:250, ab16667, Abcam) at 4 °C. After washing with PBS, the cells were incubated with anti‐rabbit IgG (1:500, Alexa Fluor 555 Conjugate, 4413, CST) for 1 h at room temperature. The nucleus was marked with DAPI, and the cover slides were mounted under dark conditions and visualized using CLSM. Ki67 positive cells and total cells were separately counted using ImageJ software based on CLSM images. The Ki67 positive cells were calculated as Ki67 positive ratio (%) = Ki67 positive cells/total cells × 100%.

### Laser‐Induced CNV Mouse Model

The healthy adult male C57BL/6J mice (6‐8 weeks old) used in the laser‐induced CNV research were given by Shanghai Jesjie Laboratory Animal Co., LTD. Mice were housed in an environmentally controlled room (23·c. with 55 ± 5% humidity and a 12 h/12 h light‐dark cycle). All tests were approved by the Eye, Otolaryngology Hospital attached to Fudan University's Animal Ethics Committee in accordance with a statement made by the Association for Research in Vision and Ophthalmology (ARVO) (Number: IRBEENT‐20210301b). A laser‐induced CNV mouse model utilizing well‐established experimental methods was developed. The mice were initially put to sleep using avodin, and then their pupils were dilated with 1% topicamide (Bausch & Lomb, China). The cornea was treated with ofloxacin eye ointment (Sinqi, China) to keep it moist when the pupil had fully dilated after 3 to 5 min. For each eye, complete 4 around the optic nerve. Each eye had four rounds of laser photocoagulation around the optic nerve while avoiding the main retinal blood vessels (532 nm, 250 mW, 50 ms duration, 50 µm fixed diameter). The idea's viability was shown by the development of bubbles during laser photocoagulation, which was viewed as a rupture of the Bruch membrane.

### Intravitreal Injection

To inhibit local reflexes brought on by the injection, a drop of topical anesthetic was administered to the eye drops before the mice were transferred to anatomical microscopy. To enlarge the pupil, mix 1–2 drops of 1% deformamide into the eyedrops that will be injected. With the eyeball stabilized with tweezers, a pre‐incision behind the limbus was made, and a needle containing medication was inserted at a 60‐degree angle behind the optic nerve, ≈1 mm into the vitreous cavity. It was crucial to remember that the lens takes up the majority of the vitreous cavity, so caution should be taken to prevent piercings. After 7 days, mice were separated into 8 groups at random: 1) PBS, 2) CNV, 3) ranibizumab, 4) LYTAC Plus, 5) LYTAC gel, 6) DNA gel, 7) scramble/DNA gel, and 8) siRNA/DNA gel.

### Fundus Fluorescein Angiography (FFA)

One week after intravitreal injection, the CNV leaking location was found using fundus fluorescein angiography (FFA). Real‐time FFA images were taken after 0.02 ml of a 10% sodium fluorescein (Alcon) solution was injected intraperitoneally.

### Choroidal‐Retinal Flat Mount and Immunostaining

After receiving therapy for a week, the male C57BL/6J mice's eyeballs were removed, immediately frozen in 4% paraformaldehyde for an h, and then put to sleep with Avodin. The RPE‐choroid‐sclera complex was formed into a flat mount using four radial incisions, which were subsequently sealed for 1 h with 10% goat serum (including 0.03% Triton X‐100) + broken membrane. After being thoroughly cleaned, the tissue was incubated with isolectin IB4 (1:50) at 4 °C overnight before being cut out. The RPE, choroid, and sclera complex were cleaned, mounted flat, and covered. ImageJ estimated the relative CNV area [(CNV area)/(total choroid area)] using IB4 pictures as a quantitative indicator.

### Optical Coherence Tomography (OCT)

OCT images were captured using Spectralis (Heidelberg, Germany). The direction of the long axis was consistent with the level of the RPE; the spindle‐shaped hyper reflexes were CNV lesions. The lesion's thickness and length were measured using a scan through the lesion's center.

### Electroretinography (ERG)

Each group of mice underwent a 12‐h dark acclimation period prior to the ERG recording. The mice's eyes were subsequently dilated and administered anesthetic. The ground electrode and the reference electrode were positioned beneath the skin of the nose and the thigh, respectively. A pair of 3 mm gold S9 ring electrodes were implanted on the cornea according to the manufacturer's instructions to perform ERG stimulation and recording using the Espion E3 device (diagnostics, Boxborough, MA). Rod and mixed cone/rod responses were elicited by a single flash of 10 cd × s m^−2^ under dark adaption conditions. A cone response of photoergs was produced after adaptation by a single stimulus intensity of 30 cd × s m^−2^ for 5 min under 10 cd × s m^−2^ of illumination. After averaging the signals for analysis, the amplitudes were provided using the Espion E3 instrument (Diagnostics, Boxborough, MA).

### TUNEL Detection

Terminal deoxynucleotidyl transferase‐mediated dUTP‐biotin nick end labeling (TUNEL) was used to evaluate safety risks in the medication group. After being fixed for 15 min using eyeball fixation solution, the frozen eye tissue pieces were cleaned three times with PBS. Then, freshly made 0.1% Triton X‐100 was used to penetrate the eye tissues, and they were exposed to a terminal deoxynucleotide transferase solution for 60 min at 37 °C. The sections were finally counterstained with the nucleic acid stain DAPI (Sigma‐Aldrich, D9542) in PBS and imaged using a laser scanning confocal microscope under 40× oil‐immersion objection lenses (TCS SP8; Leica Microsystems).

### Immunofluorescence Analysis of Mouse Retina

The mouse retinal tissue was removed, and frozen sections were made. The sections were left at room temperature for 30 min and then washed with PBS buffer at 37 °C for thrice. The sections were fixed with 4% PFA for 20 min and then washed with PBS buffer at room temperature for thrice. The sections were permeabilized with 0.1% Triton X‐100 at 37 °C for 15 min. After washing with PBS buffer, the tissue sections were blocked in 3% BSA solution.

After blocking, the sections were incubated with primary antibodies (VEGFR‐2, 1:100; ANG‐2, 1:100; RPE65, 1:50; recoverin, 1:10; PKC‐alpha, 1:50) overnight at 4 °C. Next, sections were washed 3 times with PBS buffer and dried. The sections were then incubated with fluorescent secondary antibody (anti‐rabbit 555, 1:500; anti‐mouse 488, 1:500) at room temperature for 1 h. After washing with PBS buffer, the sections were stained for nuclei using DAPI and sealed. Proteintech Anti‐VEGFR2 Polyclonal, Catalog # 26415‐1‐AP; Proteintech Anti‐ANG2 Polyclonal, Catalog # 11992‐1‐AP; Proteintech Anti‐RPE65 Polyclonal, Catalog # 17939‐1‐AP; Proteintech Anti‐Recoverin Polyclonal, Catalog # 10073‐1‐AP; Proteintech Anti‐PKC alpha Polyclonal, Catalog # 21991‐1‐AP.

### In Vivo Biodistribution Assay

Cy5‐labeled LYTAC Plus was prepared by using Cy5‐labeled linker‐2 and used to study the biodistribution in *vivo*. At days 1, 3, and 7 after vitreous injection of LYTAC Plus, the mice were sacrificed and major tissues were dissected and rinsed with PBS solution. The fluorescent images of tissue dissections were immediately recorded using the IVIS Lumina II in vivo imaging system (Caliper Life Sciences, USA). Blood was collected at the same time intervals and centrifuged at 10 000 rpm for 15 min to obtain the serum. Then, the fluorescence intensity of serum was detected by using BioTek.

### Statistics Analysis

Data were presented as mean ± SEM. Student's t test was used to compare 2 groups of samples. The difference among multiple groups was evaluated using a one‐way analysis of variance (ANOVA) with Bonferroni's post hoc test. *p < 0.05 was considered as statistically significant.

## Conflict of Interest

The authors declare no conflicts of interest.

## Author Contributions

Y.H., X.Z., and Y.Z. contributed equally to this work. C.Z. and J.H. supervised the project. Y.H., and C.Z. designed the LYTAC Plus materials. Y.H. and X.Z. performed the experiments. J.Q. contributed to the analysis of the chemical synthesis data of M6P‐N_3_. M.X. and F.W. contributed to the in vitro bioassays, and Y. Zhang, H.Y. and H.Z. contributed to the in vivo evaluation. Y.H. and X.Z. wrote the draft manuscript. Y.H., X.Z., C.Z., and J.H. discussed the results and wrote and edited the manuscript.

## Supporting information

Supporting Information

## Data Availability

The data that support the findings of this study are available in the supplementary material of this article.
