# Supplementary material for: A Nucleic Acid‐Based LYTAC Plus Platform to Simultaneously Mediate Disease‐Driven Protein Downregulation
Source: Adv Sci (Weinh). 2024 Jan 22;11(13):2306248. doi: 10.1002/advs.202306248 (PMC10987141; doi:10.1002/advs.202306248)
Supplement: Supplementary file 1 — Supporting Information [file ADVS-11-2306248-s001.pdf]

ADVANCED SCIENCE
Open Access

Supporting Information

for *Adv. Sci.*, DOI 10.1002/advs.202306248

A Nucleic Acid-Based LYTAC Plus Platform to Simultaneously Mediate Disease-Driven Protein Downregulation

Yangyang Huang, Xujiao Zhou, Yirou Zhang, Miao Xie, Fujun Wang, Jingcan Qin, Han Ye, Hong Zhang, Chuan Zhang and Jiaxu Hong**

Supporting Information

A Nucleic Acid-Based LYTAC Plus Platform to Simultaneously Mediate Disease-Driven Protein Downregulation

Yangyang Huang†, Xujiao Zhou†, Yirou Zhang†, Miao Xie, Fujun Wang, Jingcan Qin, Han Ye, Hong Zhang, Chuan Zhang, and Jiaxu Hong**

† These authors contributed equally to this work.

Experimental section

Materials.

DNAs, siRNAs, polyacrylamide gel, and agarose were purchased from Sangon Biotech (Shanghai) Co., Ltd. VEGF pepetide-N_3 ($VEGF_{125–136}$, N_3-C_6-PEG_3-QKRKRKKSRYKS[1]) was synthesized and purchased from GL Biochem (Shanghai) Ltd. All other reagents and reagent-grade solvents were purchased from Tansoole Co., Ltd (China). D-mannose, 3-bromo-1-propanol, sodium methoxide, and Di-tert-butyl N, N-diisopropyl-phosphoramidite were purchased from Macklin Biochemical Technology (Shanghai) Co., Ltd. DMAP, trityl chloride, formic acid, and 1H-tetrazole, were purchased from Beijing InnoChem Science & Technology Co., Ltd. Boron trifluoride etherate was obtained from J&K Scientific (Shanghai) Ltd. Sodium azide was purchased from Greatwall Biochemical (Wuhan) Co., Ltd. Acetic anhydride, Dowex 50WX2, diethyl ether, triethylamine, and trifluoroacetic acid were acquired from Sigma-Aldrich. DBCO-NHS was purchased from yuanye Bio-Technology (Shanghai) Co., Ltd.

Synthesis of M6P-N_3

1) Synthesis of M1

D-mannose (5 g, 27.75 mmol) was dissolved in anhydrous pyridine (25 mL), and acetic anhydride (26.06 mL, 277.5mmol) was added to dissolve the mannose at room temperature. Then DMAP (1.70 g, 13.91 mmol) was added into the system and stirred for 24 hours. After the reaction, pyridine was removed by rotary evaporation, and

ethyl acetate was added to dissolve the product. Next, the organic solution was washed with hydrochloric acid solution (0.1 M), saturated $NaHCO_3$, and distilled water respectively. After drying with Na_2SO_4 and concentrated by rotary evaporation, the residue was purified by flash chromatography on silica gel (petroleum/ethyl acetate = 1:3) to give a colorless oil (M1, 10.28 g, 95%).

2) Synthesis of M2

M1 (10.0 g, 25.6 mmol) and 3-bromo-1-propanol (4.62 g, 33.3 mmol) were successively added to 25 mL of anhydrous dichloromethane, and then 30 mL of Boron trifluoride etherate was slowly added at ice bath. After 0.5 h, the ice bath was removed and the mixture solution continue to stir for 24 h at room temperature. Finally, the reaction was quenched by saturated $NaHCO_3$, the organic phase was washed with saturated $NaHCO_3$ and distilled water (twice), dried with anhydrous Na_2SO_4, and concentrated by a rotary evaporator. The pure product (M2) was obtained by the purification of flash column chromatography (ethyl acetate /petroleum ether = 1:3, v/v)　Yield：35%.

3) Synthesis of M3

M2 (2.69 g, 5.75 mmol) was dissolved in 10 mL of dry dimethylformamide, and sodium azide (1.86 g, 28.75mmol) was added slowly and carefully. Then the mixture solution was stirred at 80˚C for 24 h. After cooling, the insoluble object was filtered and the filtrate was concentrated by a rotary evaporator, and the residue was redissolved in ethyl acetate. Then the organic phase was washed with distilled water (twice), dried with anhydrous Na_2SO_4, and evaporated by a rotary evaporator. The product (M3) was got without further purification. Yield：100%.

4) Synthesis of M4

M3 (0.797 g, 1.85 mmol) was dissolved in 10 mL of dry methanol, and sodium methoxide (0.12 g, 2.22 mmol) was added slowly. And then the mixture solution was stirred at 0˚C for 4 h. The pH of the solution was adjusted to 6 by Dowex 50WX2, H form, ion-exchange resin. The ion-exchange resin was filtered and the filtrate was

concentrated by a rotary evaporator to afford M4 without further purification. Yield: 95 %.

5) Synthesis of M5

M4 (0.789 g, 3 mmol), trityl chloride (4.17 g. 15 mmol), and DMAP (36.3 mg, 0.3 mmol) were successively added to 10 mL of pyridine, and then the mixture solution was stirred at 55˚C for 24 h. After cooling, the solvent was removed by a rotary evaporator, the residue was redissolved in dichloromethane was washed with 0.1 M hydrochloric acid solution, saturated $NaHCO_3$, and distilled water (twice), dried with anhydrous Na_2SO_4 and evaporated by a rotary evaporator. The crude product was purified using flash column chromatography (ethyl acetate /dichloromethane = 1:1, v/v) to get the yellow oil (M5). Yield: 40 %.

6) Synthesis of M6

1.2 mL acetic anhydride was slowly added to 10 mL of pyridine containing M5 (0.55 g, 1.1 mmol) at 0˚C. And then the mixture solution was stirred at room temperature for 24 h. The residue was redissolved in dichloromethane following that the solvent was removed. The organic phase was washed with 0.1M hydrochloric acid solution, saturated $NaHCO_3$, and distilled water (twice), dried with anhydrous Na_2SO_4, and evaporated by a rotary evaporator. The oil product (M6) was obtained by purification using flash column chromatography (elution: dichloromethane). Yield: 81 %.

7) Synthesis of M7

3 mL of dichloromethane containing M6 (0.631g, 1 mmol) was slowly added into the mixture solution of formic acid and diethyl ether (1:1~3ml/3ml) and stirred at room temperature for 1.5 h. After adding 100 mL of dichloromethane, the organic phase was washed with distilled water (twice), dried with anhydrous Na_2SO_4, and removed by a rotary evaporator. The oil product (M7) was obtained by flash column chromatography (ethyl acetate /dichloromethane = 1:1, v/v). Yield: 55 %.

8) Synthesis of M8

M7 (0.447 g, 1.15 mmol) was dissolved in 10 mL of dichloromethane, 1H-tetrazole (0.24g, 3.45mmol), 5mL of dichloromethane containing Di-tert-butyl N, N-diisopropyl-phosphoramidite (0.64 g, 2.3 mmol) were successively added and stirred at room temperature. After 1.5 h, 1.5 mL of triethylamine and 0.5-0.6 mL of 30% H_2O_2 were added, and then the mixture solution continued to react for another 1.5 h. After 100 mL of dichloromethane was added, the organic phase was washed with distilled water (twice), dried with anhydrous Na_2SO_4, and removed by a rotary evaporator. The oil product (M8) was purified using flash column chromatography by flash column chromatography (elution: ethyl acetate /petroleum ether = 1:1, v/v). Yield： 40 %.

9) Synthesis of M9

M8 (0.29 g, 0.5 mmol) was dissolved in 5 mL of dry methanol, and sodium methoxide (0.32 mg, 0.6 mmol) was added slowly. And then the mixture solution was stirred at 0˚C for 4 h. The pH of the solution was adjusted to 6 by Dowex 50WX2, H form, ion-exchange resin. The ion-exchange resin was filtered and the filtrate was concentrated by a rotary evaporator to afford M4 without further purification. Yield： 90 %.

10) Synthesis of M10

M9 (0.1g, 0.22 mmol) was slowly added to the mixture solution of trifluoroacetic acid and dichloromethane (1:1~3ml/3ml) and was stirred at 0˚C for 3 h. After the reaction finished, the mixture solution was added dropwise into amounts of diethyl ether, some sediment was observed, and the solid was separated by centrifugation. And then the solid was redissolved in methanol and separated by centrifugation, the precipitate was discarded and the supernatant was concentrated by a rotary evaporator. Yield： 65 %. 1H NMR (700 MHz, Deuterium Oxide) δ 4.88 (d, J = 1.7 Hz, 1H), 4.17 (dd, J = 11.4, 5.6 Hz, 1H), 4.15 – 4.11 (m, 1H), 3.97 (dd, J = 3.4, 1.7 Hz, 1H), 3.88 – 3.81 (m, 2H), 3.76 (d, J = 6.8 Hz, 2H), 3.61 (dt, J = 10.0, 5.9 Hz, 1H), 3.46 (h, J = 6.1 Hz, 2H), 1.92 (q, J = 5.7 Hz, 2H).

Supplementary Tables

Table S1. Sequences of nucleic acids used in this study.

Oligonucleotides	Oligonucleotide sequences (5’- 3’)
Y-a-NH_2	TGGATCCGCATGACATTCGCCGTAAGTTGACCTGTGAA-NH_2
Y-b-NH_2	CTTACGGCGAATGACCGAATCAGCCTTTGACCTGTGAA-NH_2
Y-c-NH_2	AGGCTGATTCGGTTCATGCGGATCCATTGACCTGTGAA-NH_2
Linker-1	CCTACTGGCATTTGCTGAACGCATTTTCACAGGTCAA-NH_2
Linker-2	AATGCGTTCAGCAAATGCCAGTAGGTTCACAGGTCAA
Linker-2-FAM	AATGCGTTCAGCAAATGCCAGTAGGTTCACAGGTCAA-FAM
Human-sense-siRNA (anti-ANG-2)	rArCrUrGrCrArArUrCrUrGrArCrArGrUrUrUrArUrUrCrArCrArGrGrUrCrA
Human-antisense-siRNA (anti-ANG-2)	rUrArArArCrUrGrUrCrArGrArUrUrGrCrArGrUrUrUrCrArCrArGrGrUrCrA
Human-scramble-sense-siRNA (anti-ANG-2)	rUrUrCrUrCrCrGrArArCrGrUrGrUrCrArCrGrUrUrUrCrArCrArGrGrUrCrA
Human-scramble-antisense-siRNA (anti-ANG-2)	rArCrGrUrGrArCrArCrGrUrUrCrGrGrArGrArArUrUrCrArCrArGrGrUrCrA
Mouse-sense-siRNA (anti-ANG-2)	rUrGrGrGrArGrUrUrCrArGrCrArGrUrArArArUrUrUrCrArCrArGrGrUrCrA
Mouse-antisense-siRNA (anti-ANG-2)	rArUrUrUrArCrUrGrCrUrGrArArCrUrCrCrCrArUrUrCrArCrArGrGrUrCrA
Mouse-scramble-sense-siRNA (anti-ANG-2)	rGrGrCrUrCrUrArArGrCrUrArArCrUrGrArArGrUrUrCrArCrArGrGrUrCrA
Mouse-scramble-antisense-siRNA (anti-ANG-2)	rCrUrUrCrArGrUrUrArGrCrUrUrArGrArGrCrCrUrUrCrArCrArGrGrUrCrA
Forward-primer (ANG-2)	GGGAAGGGAATGAGGCTTAC

Reverse-primer (ANG-2)	AAGTTGGAAGGACCACATGC
Forward-primer (GAPDH)	CAGGAGGCATTGCTGATGAT
Reverse-primer (GAPDH)	GAAGGCTGGGGCTCATTT

*Sequences with underline were the sticky ends of DNAs and siRNAs.

Supplementary Schemes and Figures

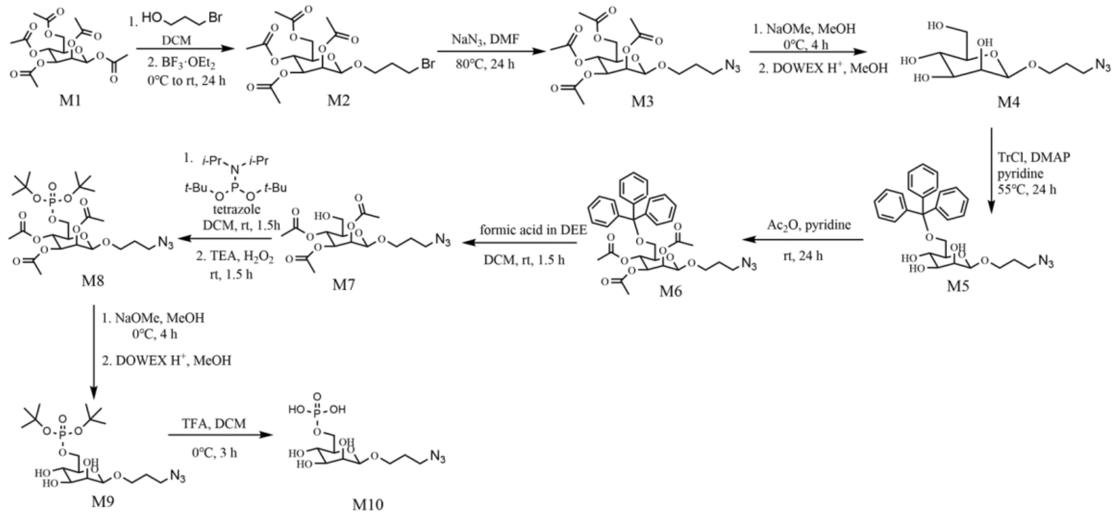

Scheme S1. The synthetic route of M6P-N_3 molecule.

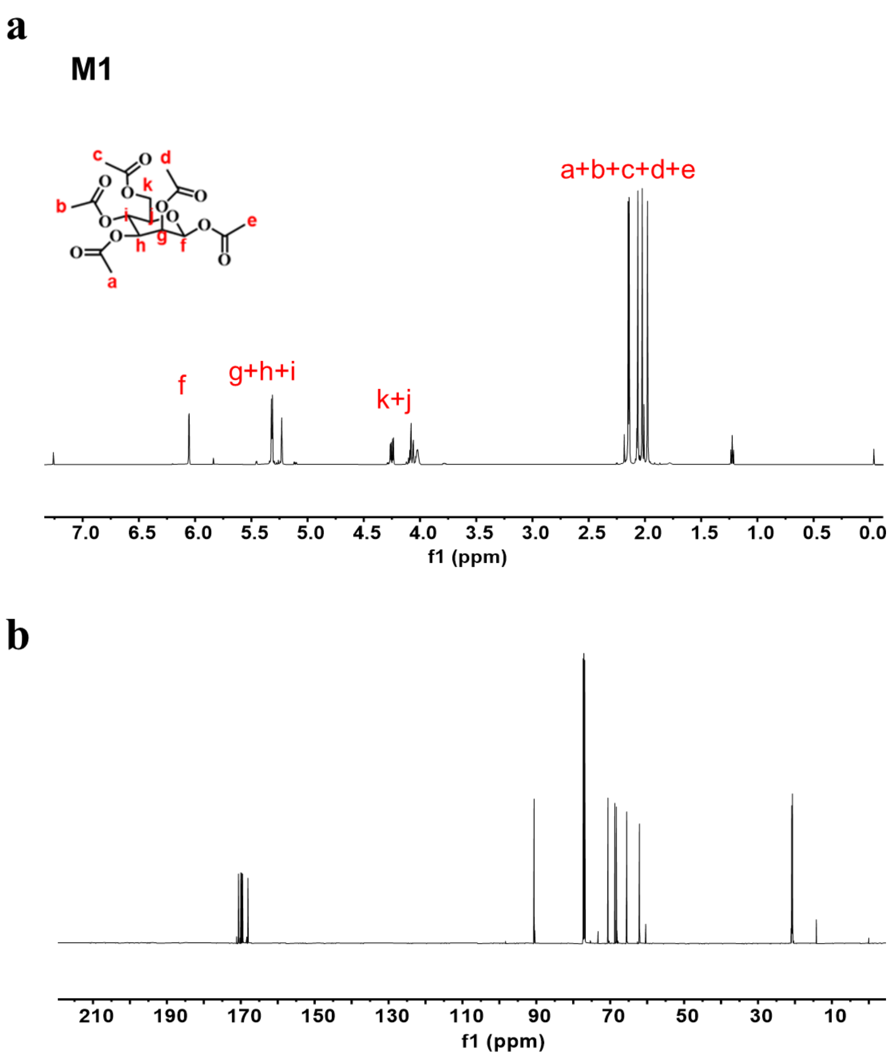

Figure S1. The ^{1}H-NMR spectrum (a) and ^{13}C-NMR spectrum (b) of M1 in $CDCl_3$.

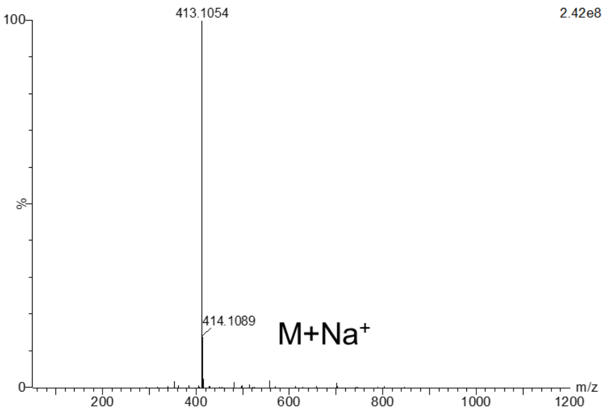

Figure S2. The mass spectrometry (MS) analysis of M1.

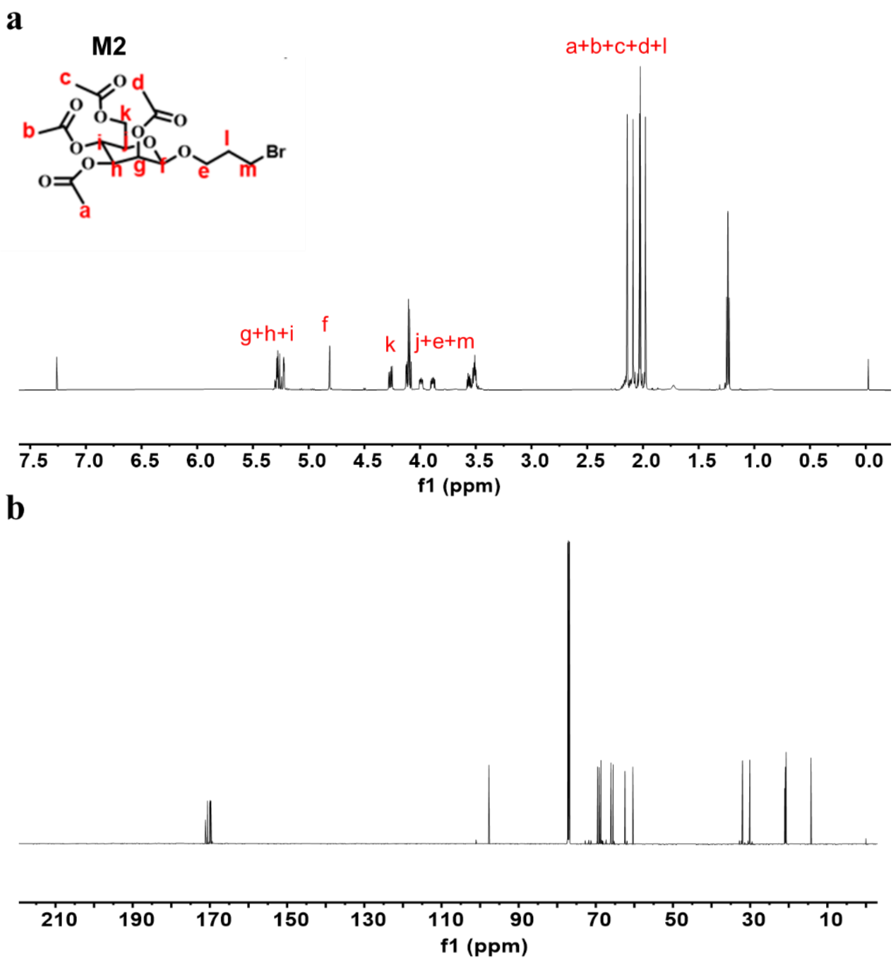

Figure S3. The ^{1}H-NMR spectrum (a) and ^{13}C-NMR spectrum (b) of M2 in $CDCl_3$.

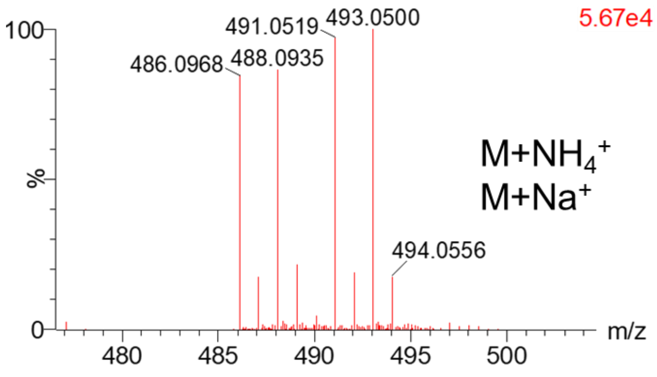

Figure S4. The mass spectrometry (MS) analysis of M2.

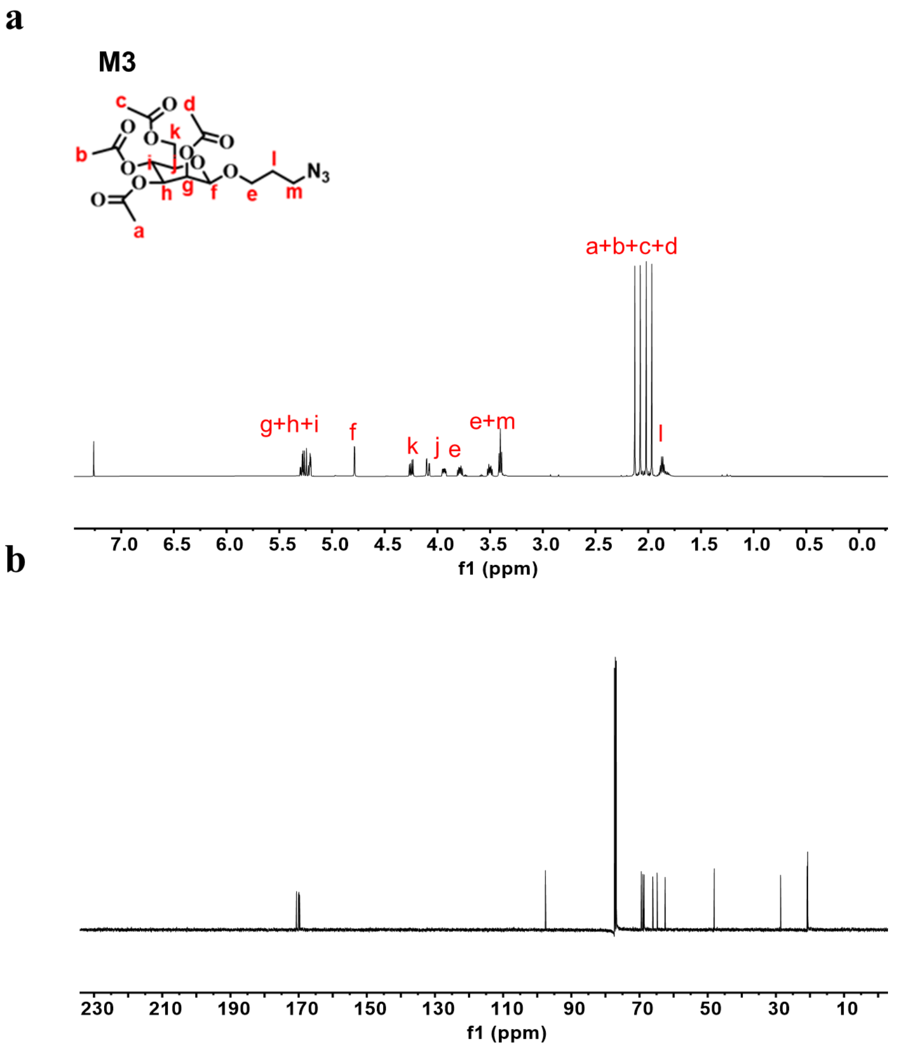

Figure S5. The ^{1}H-NMR spectrum (a) and ^{13}C-NMR spectrum (b) of M3 in $CDCl_3$.

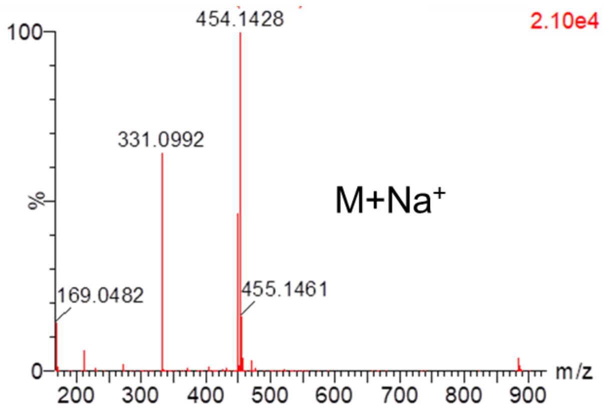

Figure S6. The mass spectrometry (MS) analysis of M3.

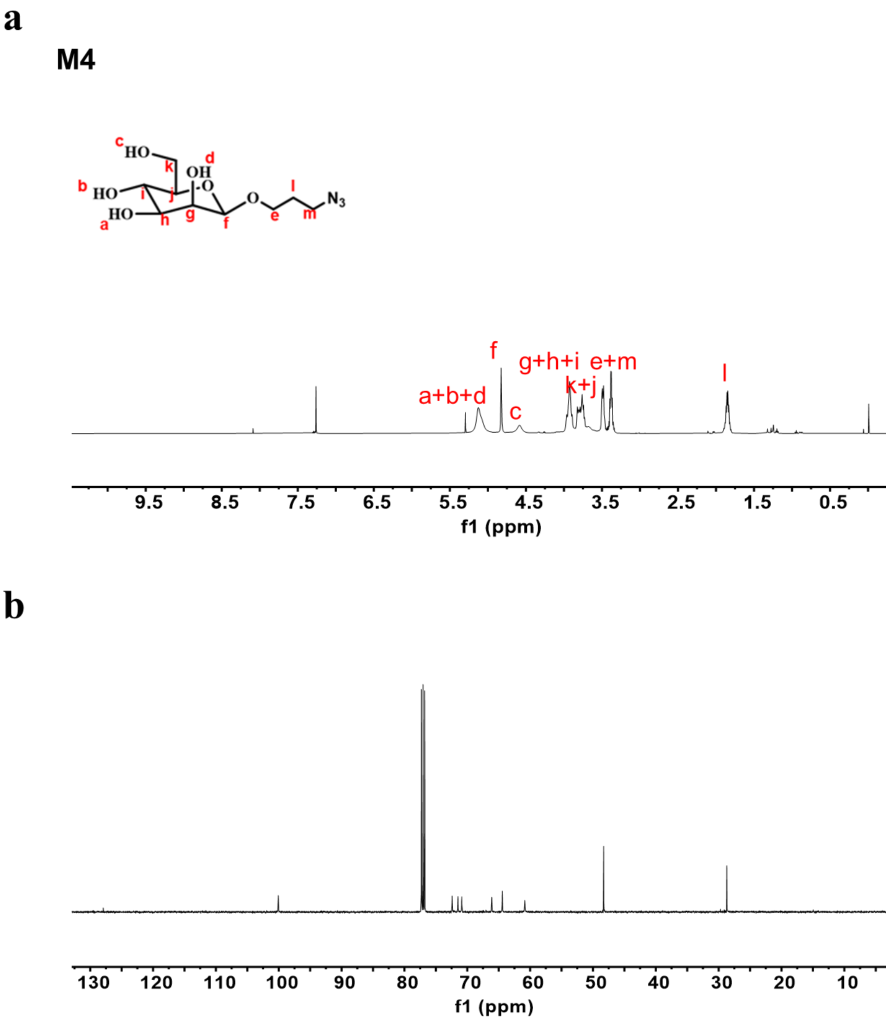

Figure S7. The ^{1}H-NMR spectrum (a) and ^{13}C-NMR spectrum (b) of M4 in $CDCl_3$.

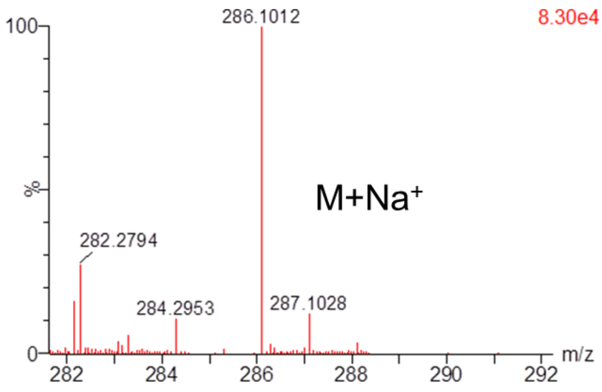

Figure S8. The mass spectrometry (MS) analysis of M4.

a

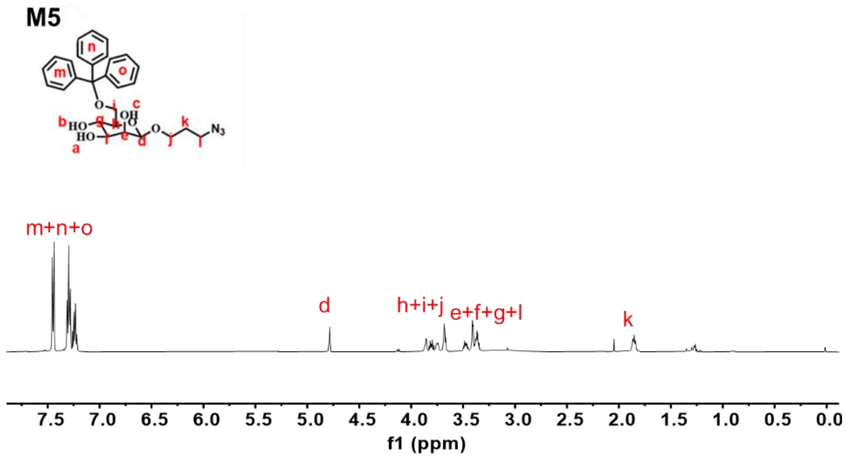

b

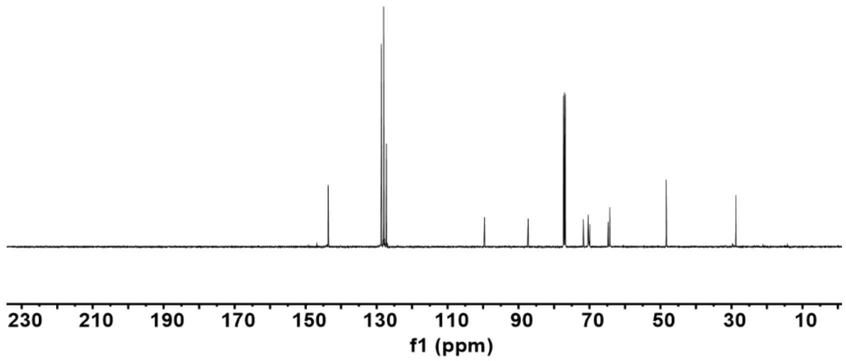

Figure S9. The ^{1}H-NMR spectrum (a) and ^{13}C-NMR spectrum (b) of M5 in $CDCl_3$.

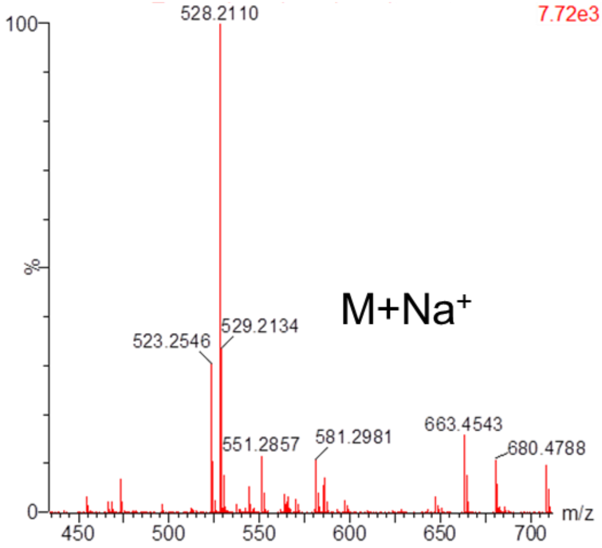

Figure S10. The mass spectrometry (MS) analysis of M5.

a

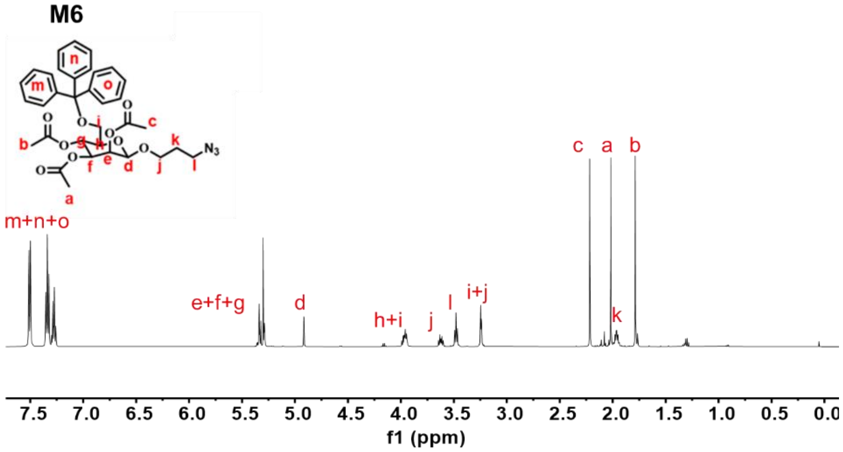

b

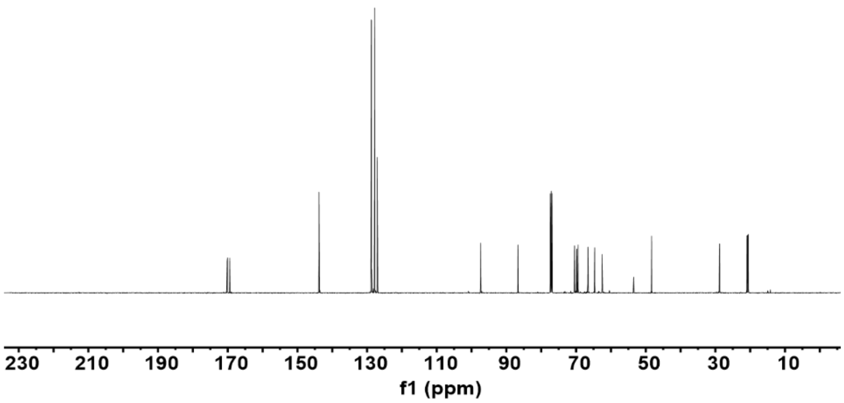

Figure S11. The ^{1}H-NMR spectrum (a) and ^{13}C-NMR spectrum (b) of M6 in $CDCl_3$.

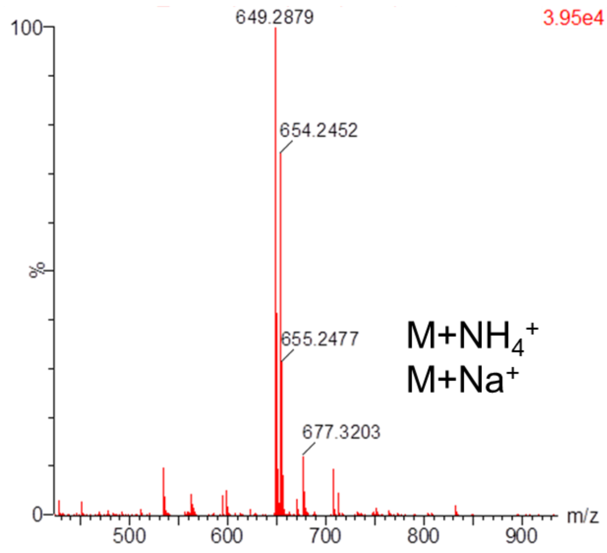

Figure S12. The mass spectrometry (MS) analysis of M6.

a

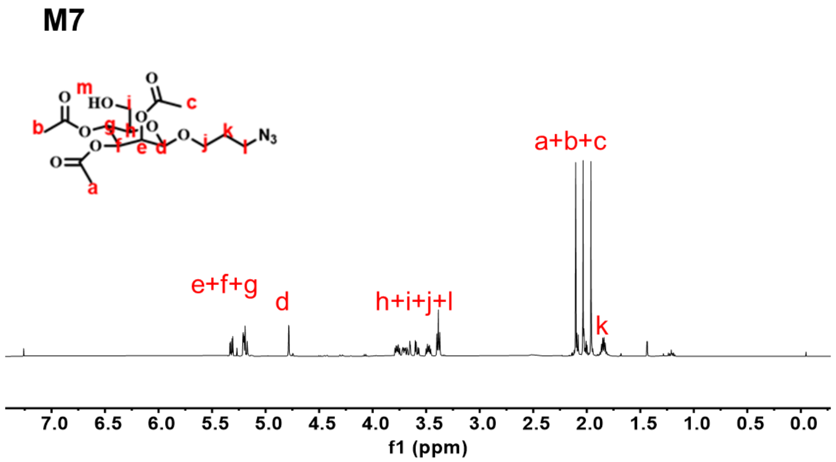

b

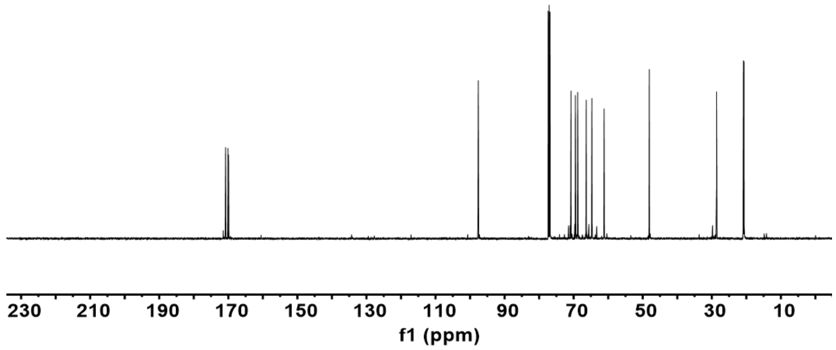

Figure S13. The ^{1}H-NMR spectrum (a) and ^{13}C-NMR spectrum (b) of M7 in $CDCl_3$.

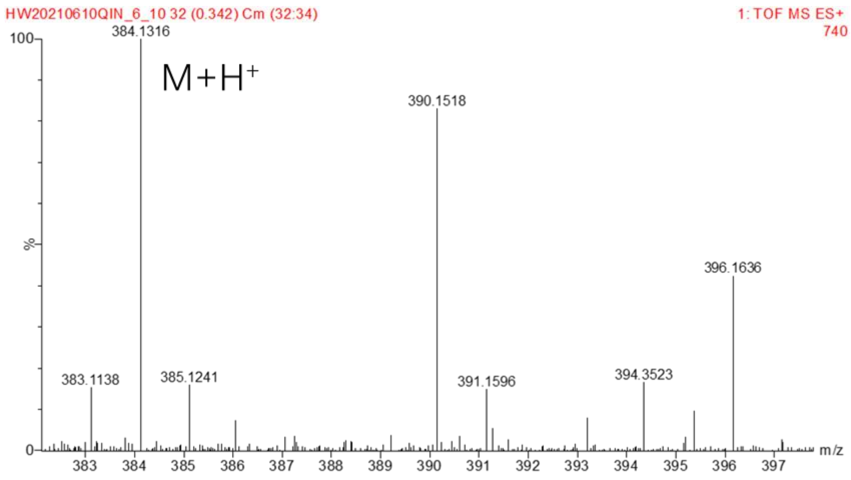

Figure S14. The mass spectrometry (MS) analysis of M7.

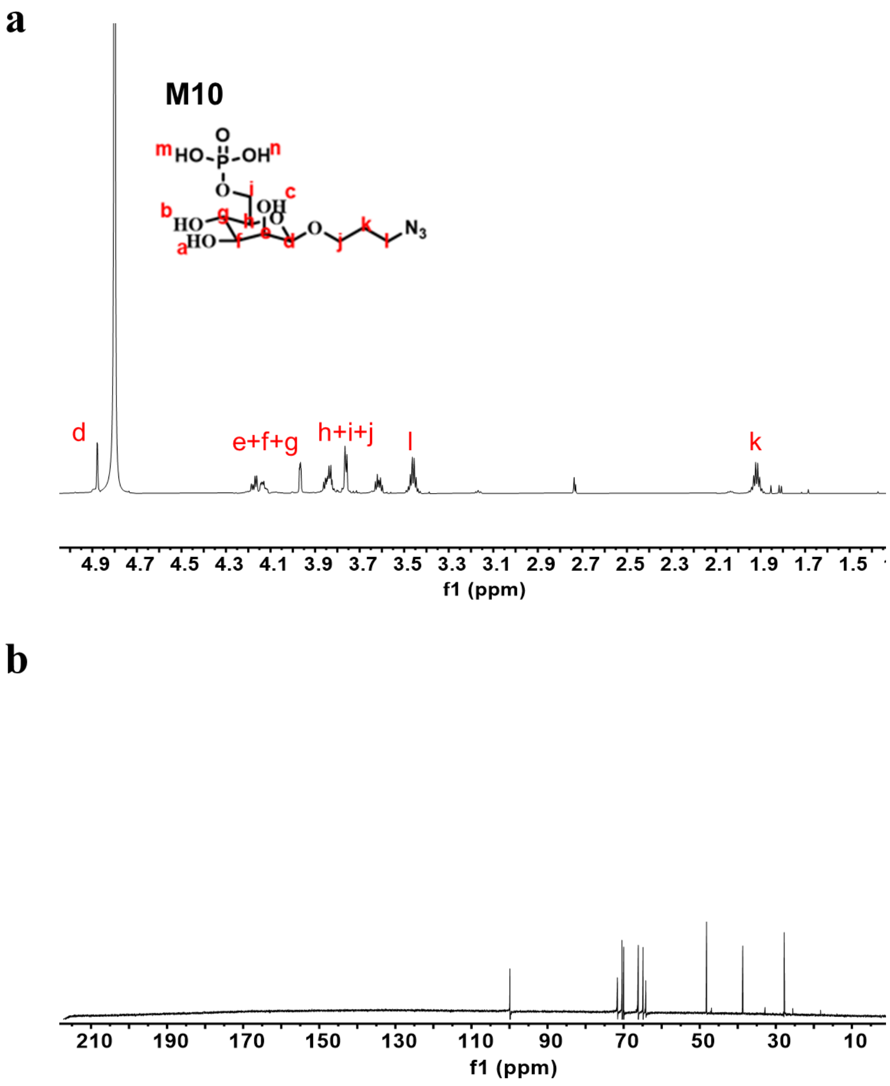

Figure S15. The ^{1}H-NMR spectrum (a) and ^{13}C-NMR spectrum (b) of M10 in $CDCl_3$.

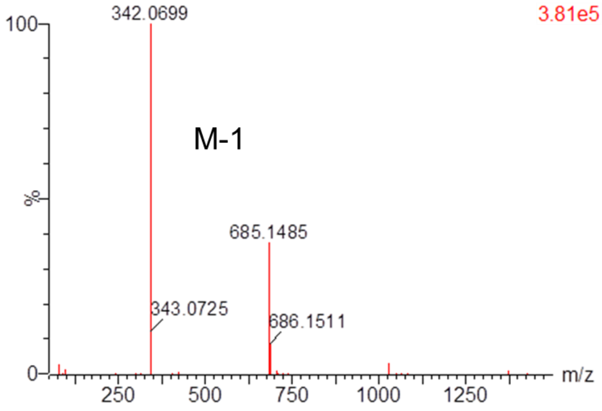

Figure S16. The mass spectrometry (MS) analysis of M10.

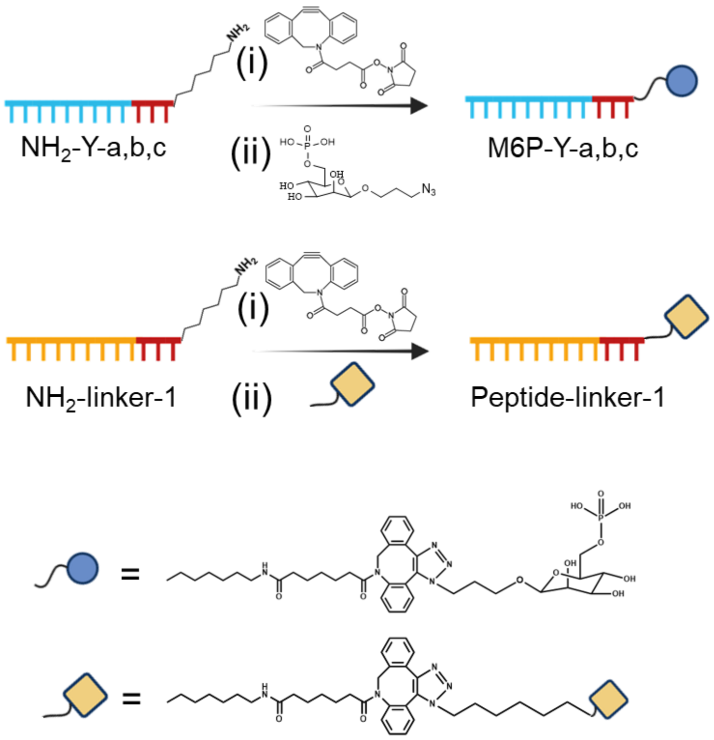

Scheme S2. The illustration of synthesis procedures of M6P or peptide-modified DNA.

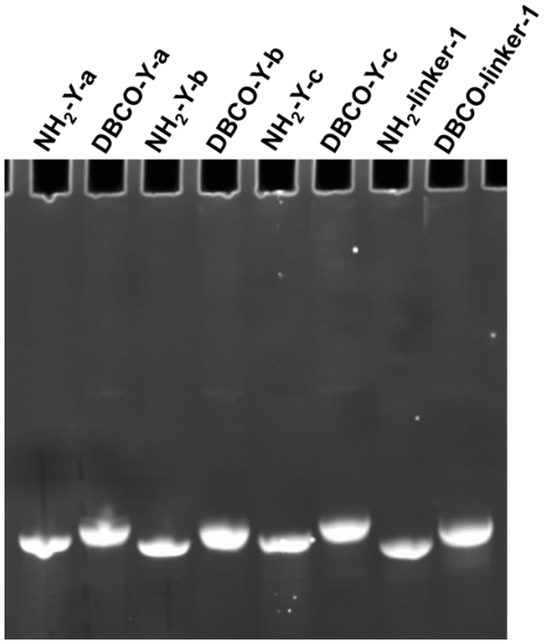

Figure S17. DBCO-modified DNAs analyzed by 20% denaturing PAGE.

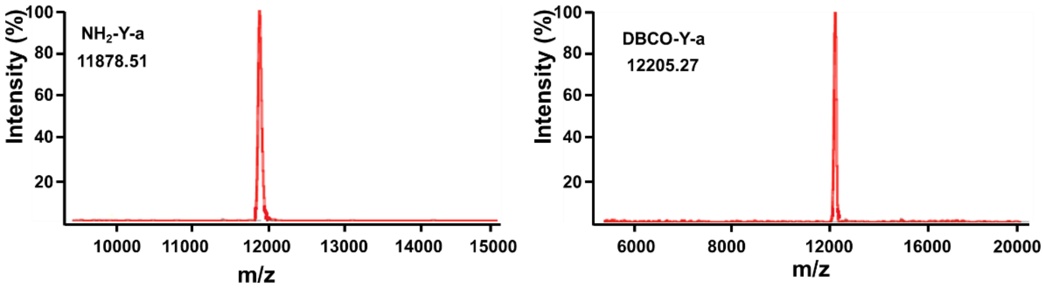

Figure S18. MALDI-TOF spectra of Y-a before and after DBCO-NHS ester modification.

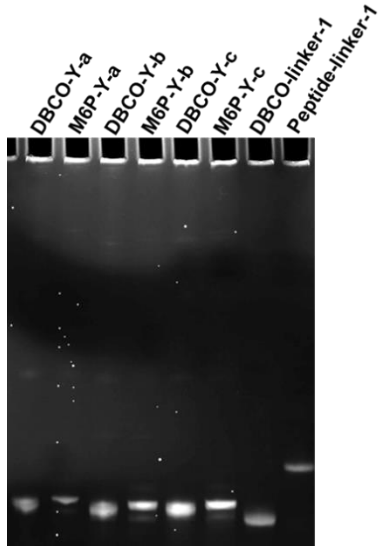

Figure S19. M6P-modified DNAs and peptide-modified DNAs analyzed by 20% denaturing PAGE.

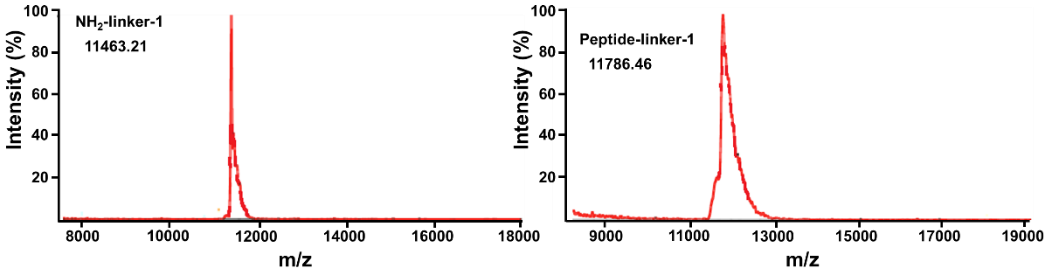

Figure S20. MALDI-TOF spectra of linker-1 before and after DBCO-NHS ester modification.

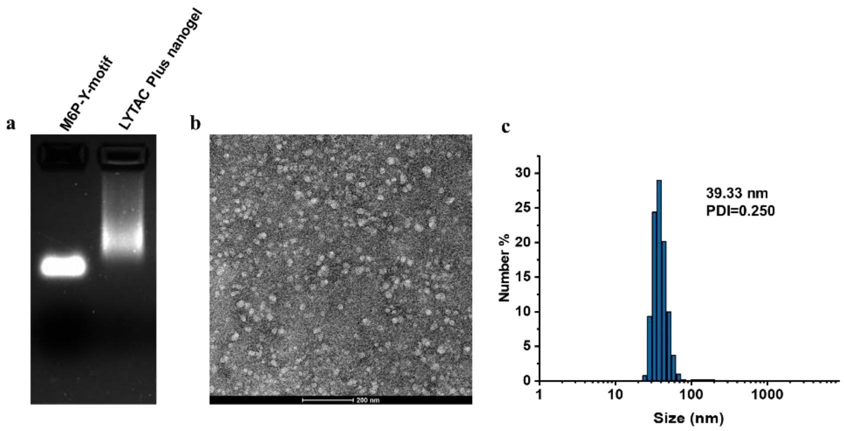

Figure S21. Characterizations of LYTAC Plus nanogel. The LYTAC Plus nanogel analyzed by (a) 1% agarose gel under native condition, (b) TEM and (c) dynamic light scattering (DLS) analysis.

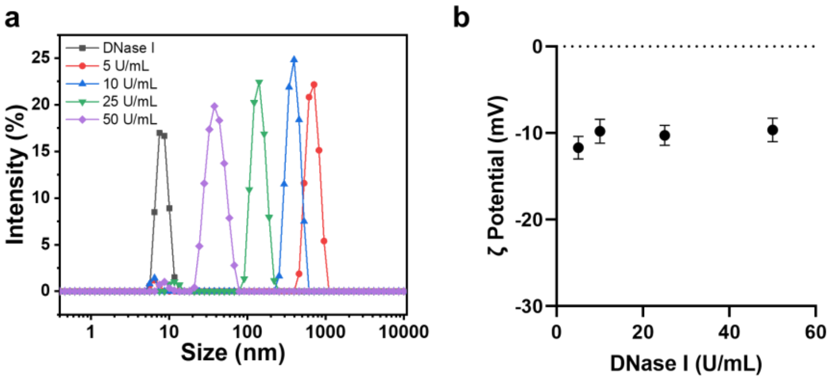

Figure S22. (a) DLS and (b) ζ-potential measurements of LYTAC Plus gel (400 μM in term of component Y-motifs) after incubation with different concentrations of DNase I at 37 °C for 12 h.

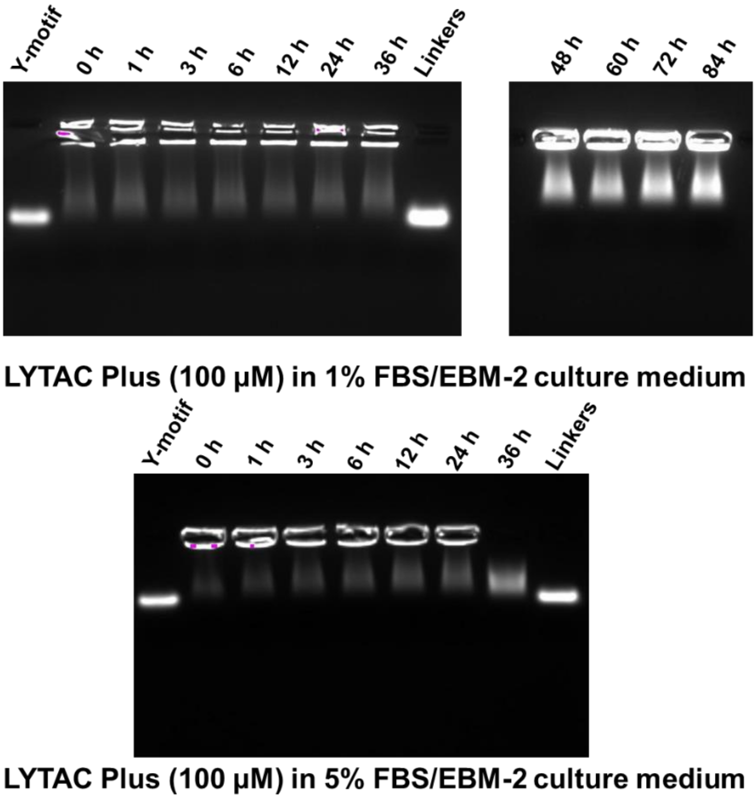

Figure S23. Disassembly of LYTAC Plus gel in 1% or 5% FBS/EBM-2 culture medium analyzed by 1% agarose under native condition.

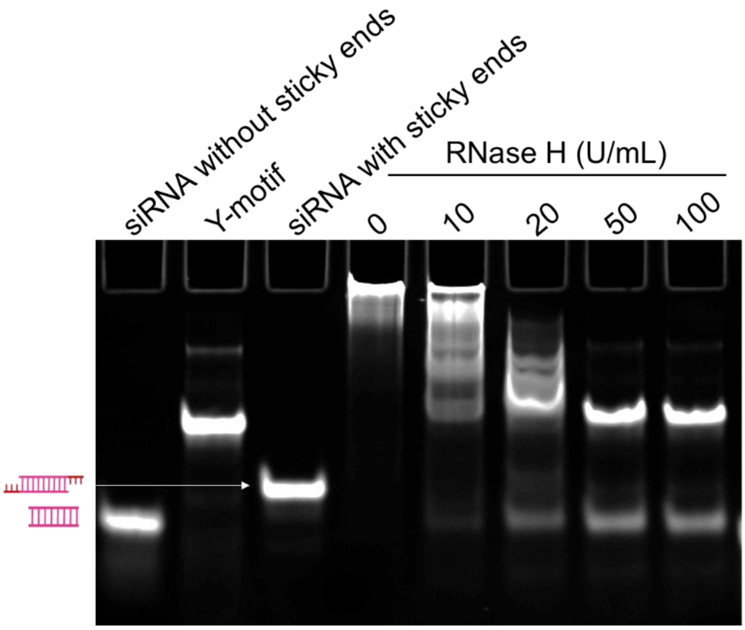

Figure S24. 10% native PAGE gel image of LYTAC Plus gel after incubation with different concentrations of RNase H for 1.0 h at 37 °C.

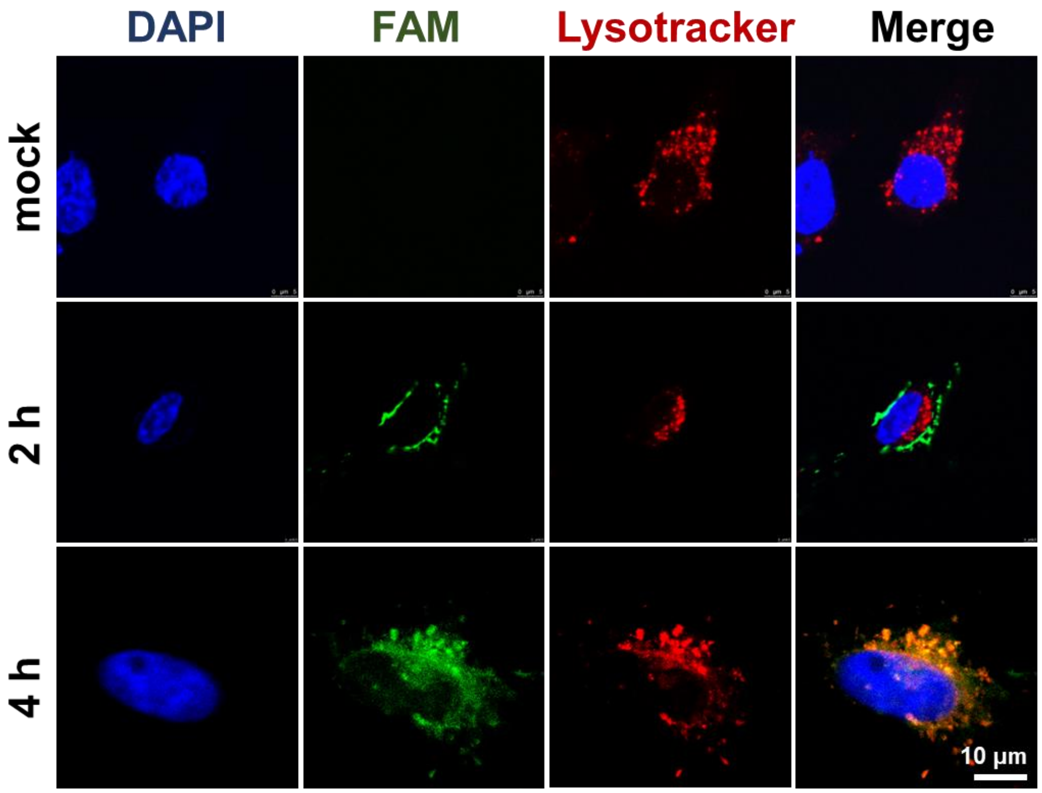

Figure S25. CLSM images of 2 μM LYTAC Plus and lysosome colocalization conditions after incubating with HUVECs for 2 and 4 h independently. Scale bar: 10 μm.

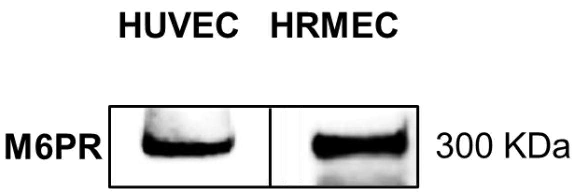

Figure S26. Western blot analysis of M6PR expression in HUVECs and HRMECs.

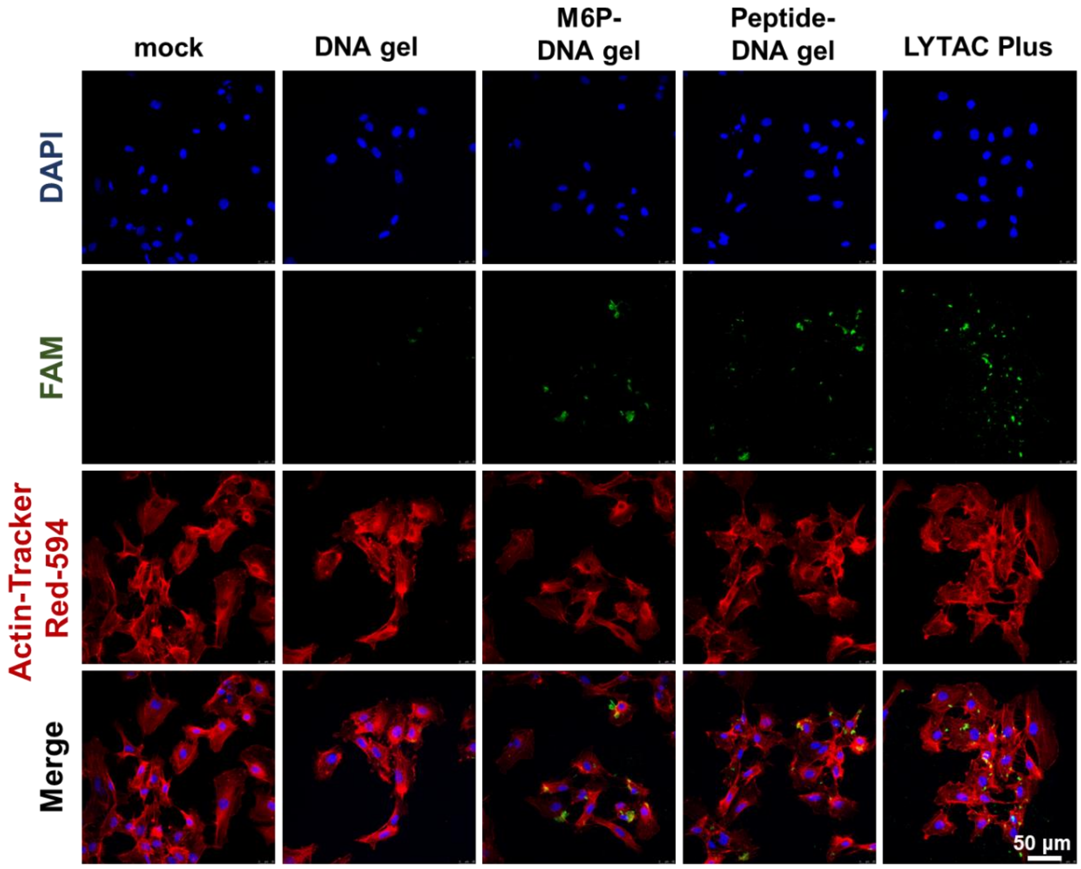

Figure S27. Confocal images of HUVECs cellular uptake after 6 h incubation with different materials (green). Scale bar: 50 μm.

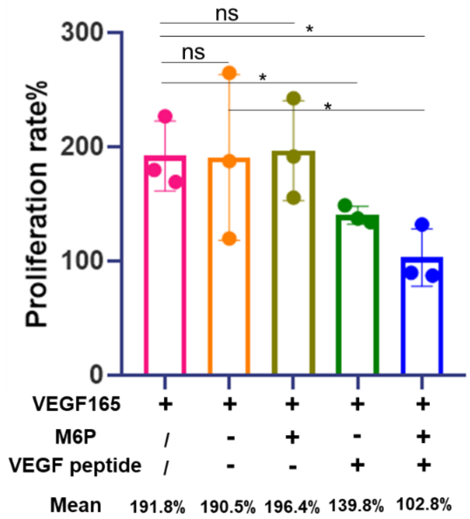

Figure S28. Cell proliferation situation of HUVECs treated with 2 μM different formations for 48 h under the stimulation of $VEGF_{165}$.

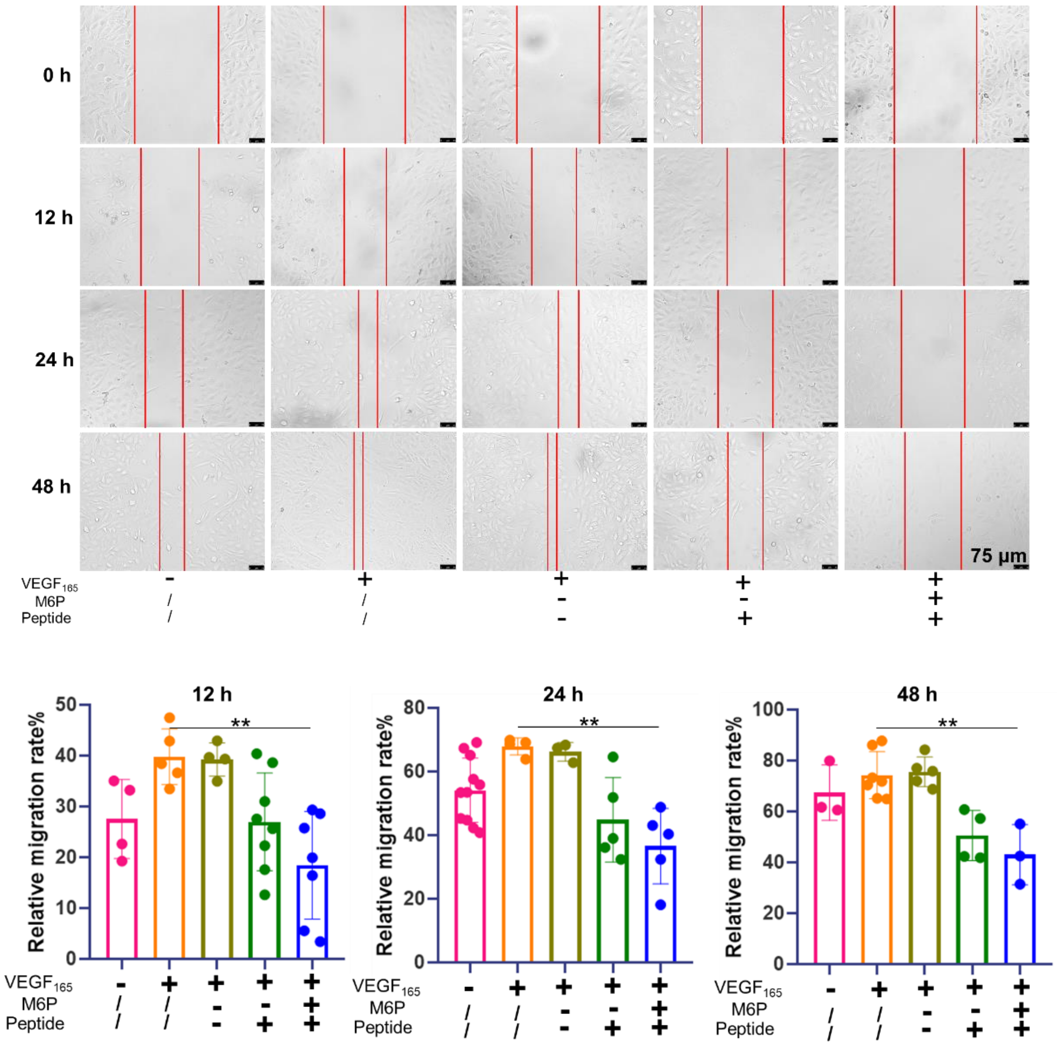

Figure S29. Cell migration capability of HUVECs after treatment with different samples and analyzed by wound-healing experiment and captured images at indicated time.

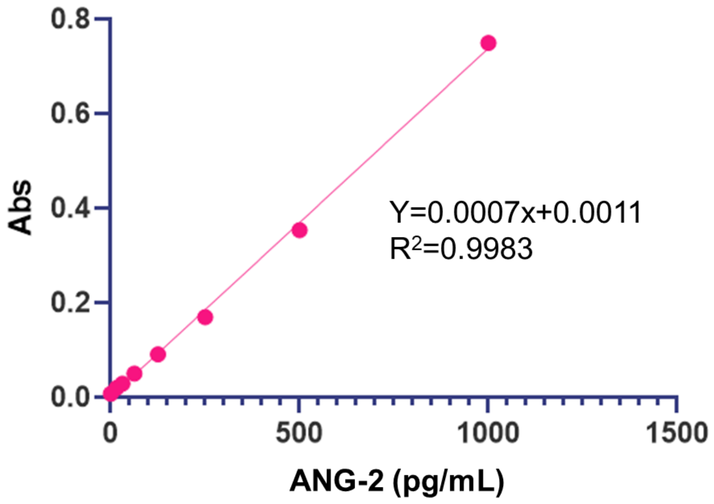

Figure S30. Standard curve of human ANG-2 ELISA kit experiment used in this study.

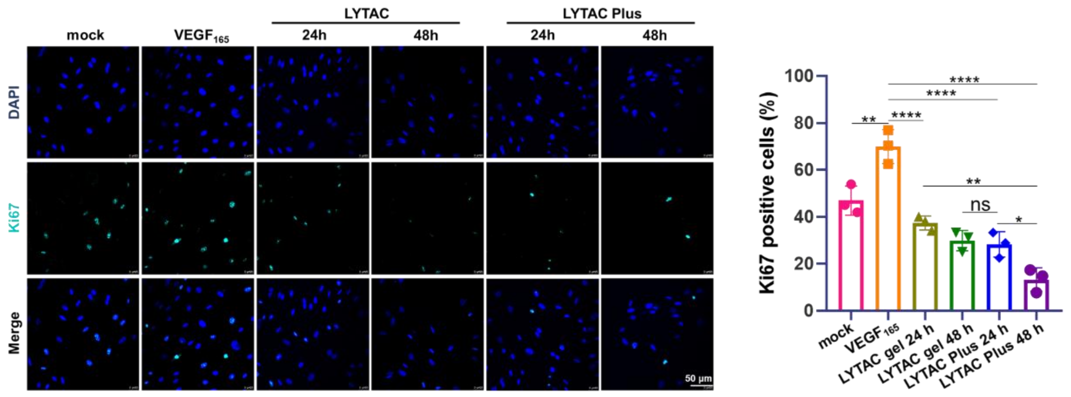

Figure S31. Immunofluorescence staining of cell proliferation marker Ki67 and Ki67-positive cell ratios of cultured HUVECs. Scale bar: 50 μm. Ki-67 positive cells and total cells were separately counted by using ImageJ software. Ki-67 positive cells (%) = Ki-67 positive cells / total cells × 100%

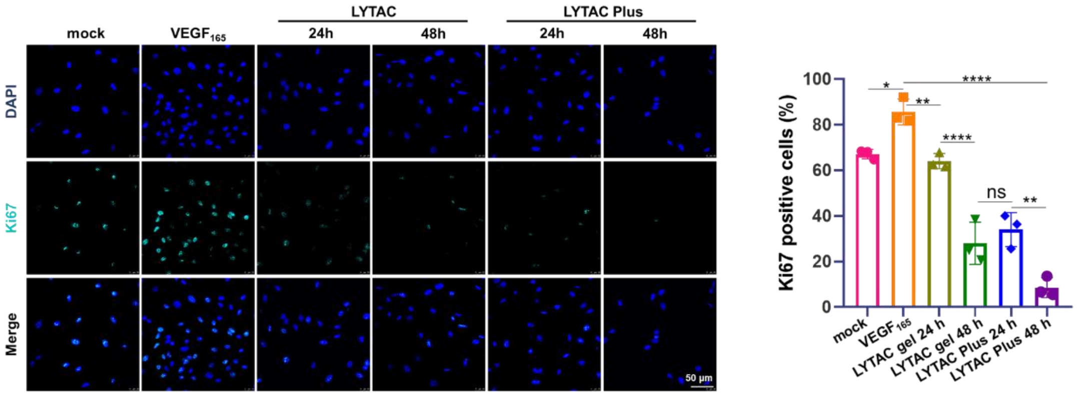

Figure S32. Immunofluorescence staining of cell proliferation marker Ki67 and Ki67-positive cell ratios of cultured HRMECs. Scale bar: 50 μm. Ki-67 positive cells and total cells were separately counted by using ImageJ software. Ki-67 positive cells (%) = Ki-67 positive cells / total cells × 100%

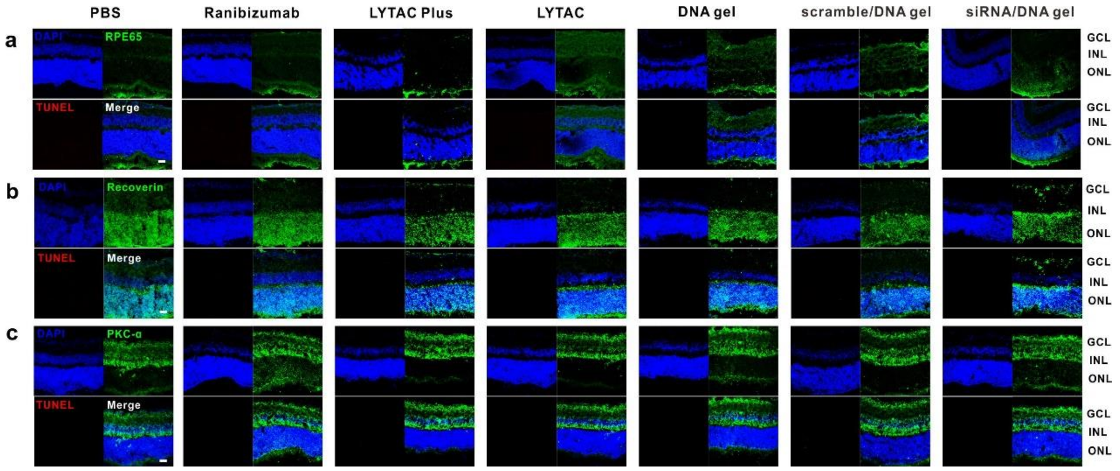

Figure S33. Immunofluorescence staining of RPE- and photoreceptor-specific markers. GCL: ganglion cell layer, INL: inner nuclear layer, ONL: outer nuclear layer. Scale bar: 50 μm.

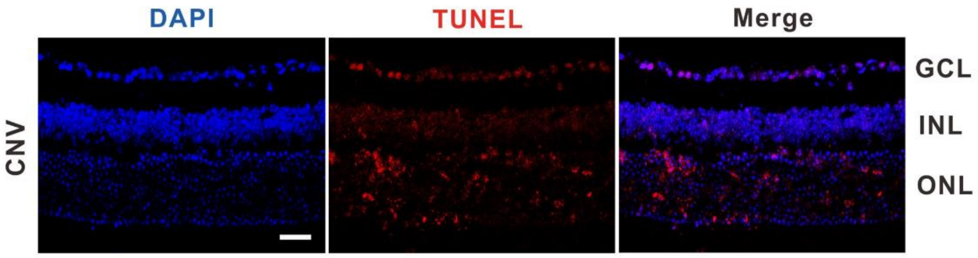

Figure S34. A representative TUNEL staining of retina from mice with lase-induced

CNV. GCL: ganglion cell layer, INL: inner nuclear layer, ONL: outer nuclear layer. Scale bar: 25 µm. N: number of eyeballs.

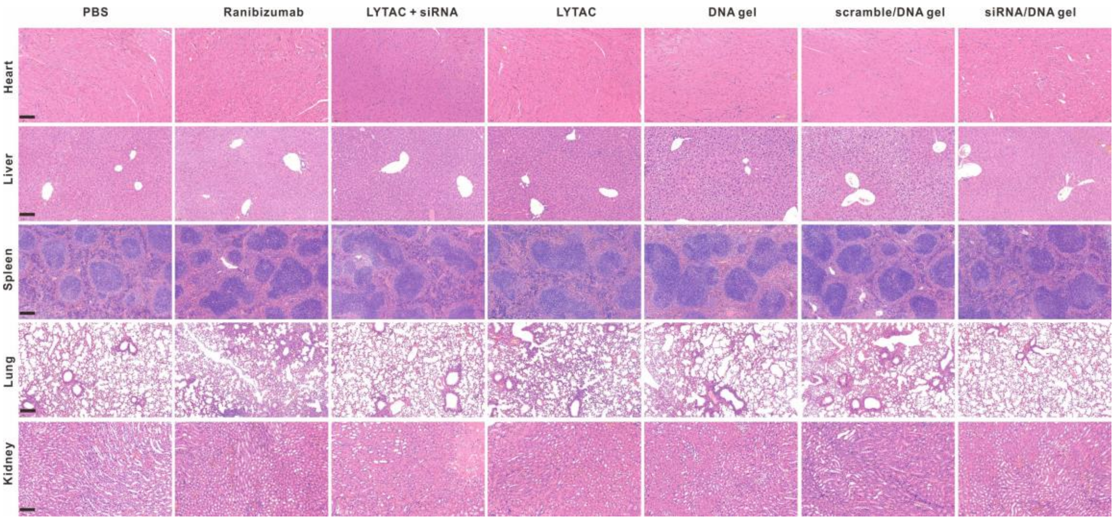

Figure S35. Systematic toxicity of designed pharmaceutical regimen. Histological staining of body tissues of mice (N' = 3) in each experimental group. Scale bar: 200 µm for spleen and lung; 100 µm for heart, liver and kidney. N', number of mice.

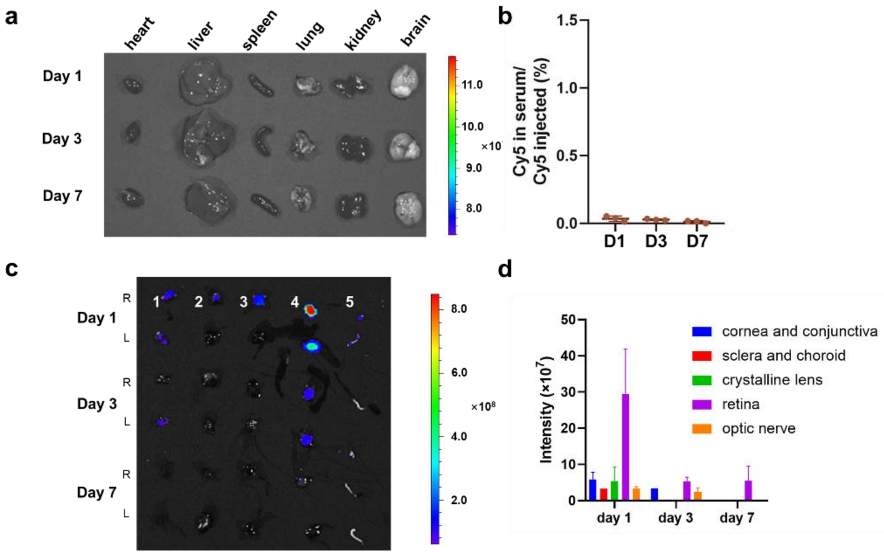

Figure S36. *In vivo* biodistribution of LYTAC Plus. Fluorescence signal of (a) main organs and (b) serum of mice within 7 days after intravitreal injection of LYTAC Plus. (c) Fluorescence imaging and (d) semi-quantitative analysis of fluorescence signals of different eye tissues of mice within 7 days after intravitreal injection of LYTAC Plus-1: cornea and conjunctiva; 2: sclera and choroid; 3: crystalline lens; 4: retina; 5: optic nerve.

Supplementary References

[1] K. Hu, J. Shang, L. Xie, M. Hanyu, Y. Zhang, Z. Yang, H. Xu, L. Wang, M. R. Zhang, *ACS Omega* **2020**, *5*, 8508.
